# Formation of Mono Oxo Molybdenum(IV) PNP Pincer Complexes: Interplay between Water and Molecular Oxygen

**DOI:** 10.1002/ejic.201701413

**Published:** 2018-02-12

**Authors:** Sara R. M. M. de Aguiar, Özgür Öztopcu, Anna Troiani, Giulia de Petris, Matthias Weil, Berthold Stöger, Ernst Pittenauer, Günter Allmaier, Luis F. Veiros, Karl Kirchner

**Affiliations:** ^1^ Institute of Applied Synthetic Chemistry Vienna University of Technology Getreidemarkt 9 1060 Vienna Austria; ^2^ Dipartimento di Chimica e Tecnologie del Farmaco Università di Roma “La Sapienza” P. le Aldo Moro 5 00185 Roma Italy; ^3^ Institute of Chemical Technologies and Analytics Vienna University of Technology Getreidemarkt 9 1060 Vienna Austria; ^4^ X‐ray Center Vienna University of Technology Getreidemarkt 9 1060 Vienna Austria; ^5^ Centro de Química Estrutural Instituto Superior Técnico Universidade de Lisboa Av. Rovisco Pais No. 1 1049‐001 Lisboa Portugal

**Keywords:** Molybdenum, Pincer ligands, Phosphines, Oxo complexes, Ligand effects

## Abstract

The synthesis of cationic mono oxo Mo^IV^ PNP pincer complexes of the type [Mo(PNP^Me^‐*i*Pr)(O)X]^+^ (X = I, Br) from [Mo(PNP^Me^‐*i*Pr)(CO)X_2_] is described. These compounds are coordinatively unsaturated and feature a strong Mo≡O triple bond. The formation of these complexes proceeds via cationic 14e intermediates [Mo(PNP^Me^‐*i*Pr)(CO)X]^+^ and requires both molecular oxygen and water. ESI MS measurements with ^18^O labeled water (H_2_^18^O) and molecular oxygen (^18^O_2_) indicates that water plays a crucial role in the formation of the Mo≡O bond. A plausible mechanism based on DFT calculations is provided. The X‐ray structure of [Mo(PNP^Me^‐*i*Pr)(O)I]SbF_6_ is presented.

## Introduction

Molybdenum complexes featuring a terminal mono oxo unit comprise an important class of compounds.^1^, ^2^ On the one hand, besides of being intrinsically interesting,^3^ such complexes are well documented to act as catalysts for various oxidation processes involving for instance molecular oxygen.^4^ They are also known to generate hydrogen from water^5^ and are applied in various catalytic reactions such as hydrosilylation^6^ and sulfur transfer to alkenes and allenes.^7^ Moreover, nature efficiently utilizes the Mo=O unit to achieve difficult multielectron redox catalysis with oxotransferases, which catalyze oxygen atom transfer to and from substrates.^8^, ^9^, ^10^ High valent Mo=O or Mo≡O species are often generated accidently by trace amounts of O_2_ or water contaminations due to the high affinity of molybdenum towards oxygen which may involve proton assisted and/or water assisted dioxygen cleavage reactions.^11^ The oxygen source of the Mo–O moiety can thus be both molecular oxygen and/or of water.

**Scheme 1:**
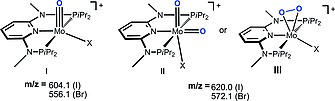
Possible oxygen‐containing species detected by fragmentation of [Mo(PNP^Me^‐*i*Pr)(CO)X_2_] (**1a, 1b**) in CH_3_CN in the presence of air and water as established by ESI MS experiments. Structural suggestions are based on DFT calculations.^12^

In keeping with the facile formation of molybdenum–oxygen bonds, we recently observed in preliminary ESI MS studies that complexes [Mo(PNP^Me^‐*i*Pr)(CO)X_2_] (X = I (**1a**), Br (**1b**)) readily form in apparently parallel pathways both mono and dioxo species which were tentatively assigned as **I** and **II** or **III** based on DFT calculations (Scheme 1).^12^ It was not clear at this stage whether the source of oxygen was molecular oxygen from air, traces of water in the solvent, or both.

We report here on a rational synthesis of cationic coordinatively unsaturated mono oxo Mo^IV^ PNP pincer complexes of the type [Mo(PNP^Me^‐*i*Pr)(O)X]^+^ (**A**) which are formed from in‐situ prepared [Mo(PNP^Me^‐*i*Pr)(CO)X]^+^ (**2a, b**) in the presence of molecular oxygen and water.^13^

## Results and Discussion

**Scheme 2:**
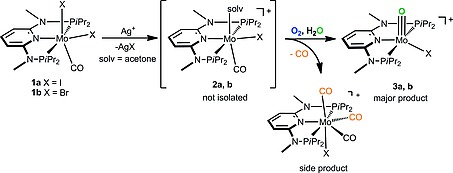
Formation of mono oxo Mo^IV^ complexes.

When a solution of [Mo(PNP^Me^‐*i*Pr)(CO)(X)(solv)]^+^ (**2a**, **b**) in acetone, prepared in situ by reacting [Mo(PNP^Me^‐*i*Pr)(CO)(X)_2_] (**1a**, **b**) (X = I, Br) with AgSbF_6_ followed by removal of AgX, is exposed shortly to air and subsequently treated with an excess of water, the cationic mono oxo complexes [Mo(PNP^Me^‐*i*Pr)(O)X]^+^ (**3a**, **b**) are afforded in 72 and 66 % isolated yields (Scheme 2). In the absence of air or water, no mono oxo complexes are formed. Accordingly, the formation of the molybdenum oxo bond requires an interplay between these two reagents. NMR and IR monitoring of the reaction with **1a** and **1b** after addition of the halide scavenger revealed the immediate formation of **2a** and **2b**, respectively. These intermediates give rise to signals at *δ* = 183.3 and 189.5 ppm in the ^31^P{^1^H} NMR spectrum and exhibit one strong ν_CO_ band at 1832 and 1840 cm^–1^, respectively (cf. 1824 cm^–1^ in **1a** and 1816 cm^–1^ in **1b**). Solvent complexes of the type [Mo(PNP^Me^‐*i*Pr)(CO)(X)(solv)]^+^ (X = Cl, Br, solv = THF, CH_3_CN) were prepared and isolated recently.^12^ Upon admission of air and addition of water, new resonances at *δ* = 149.2 and 145.2 ppm, respectively, were observed in the ^31^P{^1^H} NMR spectrum due to the formation of **3a** and **3b** and the CO stretching frequencies of **2a** and **2b** disappeared.

Complexes **3a** and **3b** were characterized by a combination of elemental analysis, ^1^H, ^13^C{^1^H}, and ^31^P{^1^H} NMR, IR and ESI MS. Characteristic are the Mo≡O stretching frequencies at 955 and 940 cm^–1^, respectively. In the ESI‐MS the most abundant signals are observed at *m/z* 604.1 and 556.1, respectively, which correspond to the intact complexes **3a** and **3b** ([M]^+^). In addition to the main products (**3a**,**b**), small amounts (ca 10 %) of the known seven‐coordinate tricarbonyl complex [Mo(PNP^Me^‐*i*Pr)(CO)_3_X]^+^ are formed as side products due to reaction of **2a** and **2b** with CO, which is released during the oxidation process (Scheme 2).^14^ It has to be noted, that there was no evidence for the formation of CO_2_ as a result of CO oxidation by O_2_.

**Figure 1:**
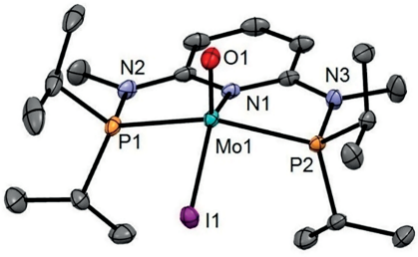
Structural diagram of [Mo(PNP^Me^‐*i*Pr)(O)I]SbF_6_ (**3a**) showing displacement ellipsoids at the 50 % probability level (hydrogen atoms and SbF_6_^–^ counterion omitted for clarity). Selected bond lengths and angles (Å, °): Mo1–O1–1.663(2), Mo1–N1 2.143(2), Mo1–P2 2.4413(8), Mo1–P1 2.4455(8), Mo1–I1 2.7359(4), O1–Mo1–N1 108.67(11), O1–Mo1–P2 104.89(8), O1–Mo1–P1–107.02(8), P2–Mo1–P1 144.81(3), O1–Mo1–I1 108.40(9), N1–Mo1–I1 142.92(6).

**Figure 2:**
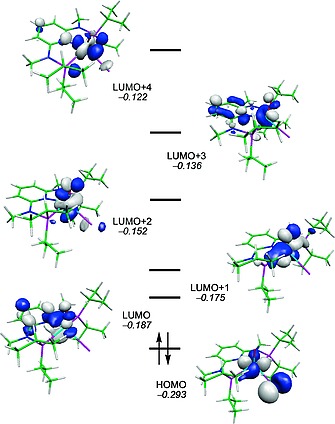
Frontier orbitals (*d*‐splitting) of [Mo(PNP^Me^‐*i*Pr)(O)I]^+^ (**3a**). Orbital energy values in atomic units.

In addition to the NMR, IR and ESI‐MS spectroscopic characterization, the crystal structure of **3a** was determined by single‐crystal X‐ray diffraction. A structural diagram is depicted in Figure 1 with selected bond lengths and angles given in the caption. Complex **3a** is best described as having a pseudo square pyramidal structure. The Mo1–O1 bond length of 1.663(2) Å is comparatively short but in the typical range for a Mo≡O triple bond.^15^, ^16^, ^17^ This has been investigated by DFT calculations.^18^ The frontier orbitals of **3a** are represented in Figure 1. The pattern obtained is typical of a d^2^ metal complex with a square pyramidal geometry.^19^ The HOMO is the *xy* orbital (the *z* axis being defined by the Mo–O bond) and the LUMO is mostly centered in the ligand pyridine ring. The two following orbitals (LUMO+1 and LUMO+2) are based on metal *yz* and *xz*, respectively (see Figure 2). Those are Mo–O π* orbitals and, thus, are the two empty antibonding counterparts of π‐donation from the oxo ligand to the metal, indicating a Mo≡O triple bond. Finally, the two upper orbitals in Figure 1 are based on the metal *z* ^2^ and *x* ^2^–y^2^.

**Figure 3:**
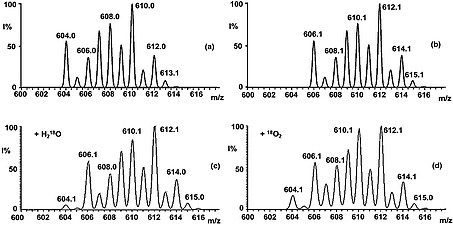
Isotopic pattern from the simulated spectra of complex **3a**: (a) [Mo(PNP^Me^‐*i*Pr)(^16^O)I]^+^ and (b) [Mo(PNP^Me^‐*i*Pr)(^18^O)I]^+^. Multiplet ions corresponding to complex **3a** formed in positive ions ESI‐MS spectra of [Mo(PNP^Me^‐*i*Pr)(CO)I_2_] (**1a**) in CH_3_CN: (c) in the presence of H_2_^18^O; (d) in the presence of ^18^O_2_.

To evaluate the role of water and O_2_ as an oxygen source, solutions of **1a** in CH_3_CN were subjected to ESI‐MS analysis in the positive ion mode in the presence of either ^18^O_2_ or H_2_^18^O (it has to be noted that traces of water and air, i.e., H_2_^16^O and ^16^O_2_, are always present in standard ESI MS experiments). An estimate of the ^18^O incorporation may be given based on the simulated spectra of the [Mo(PNP^Me^‐*i*Pr)(^16^O)I]^+^ and [Mo(PNP^Me^‐*i*Pr)(^18^O)I]^+^ (Figures 3a and 3b), showing a displacement of the multiplet towards the higher‐mass peaks at *m/z* 614–615. In the case of H_2_^18^O an approximate [Mo(PNP^Me^‐*i*Pr)(^16^O)I]^+^/[Mo(PNP^Me^‐*i*Pr)(^18^O)I]^+^ ratio of 10:90 has been found, whereas a 30:70 ratio has been found with ^18^O_2_ (Figures 3c and 3d). This finding may be taken as a circumstantial evidence of a more effective role of H_2_O compared to O_2_.

**Scheme 3:**
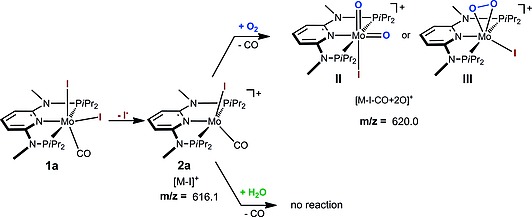
Gas phase reaction of [Mo(PNPMe‐*i*Pr)(CO)I]^+^ ions (**2a**) with ^16^O_2_ (or ^18^O_2_) and H_2_O.

In addition, we investigated the reaction of isolated [Mo(PNPMe‐*i*Pr)(CO)I]^+^ ions (**2a**) at *m/z* 616 in the gas phase with O_2_ or H_2_O. When ion **2a** is reacted with O_2_, a very slow addition of O_2_ (or ^18^O_2_) takes place forming a dioxo product ion at *m/z* 620 (or *m/z* 624) together with other products. This clearly shows that in the gas phase ion **2a** is the precursor of the dioxo species **II** or **III** as shown in Scheme 3. The same species is also formed and observed in the electrosprayed solution as already reported previously.^12^ When the ion–molecule reaction of **2a** was performed with H_2_O instead of O_2_, neither [Mo(PNPMe‐*i*Pr)(O)I]^+^ (**3a**) nor other products were observed. This suggests again the need for a cooperation between dioxygen and water that can be realized in solution (sprayed solution) but not in the gas phase where reactants and products are in a rarefied environment (pressure of about 10^–6^–10^–5^ Torr that reaches up to 10^–3^ Torr with Helium).

**Scheme 4:**
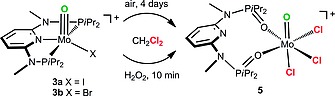
Exposure of complexes **3a** and **3b** to air or H_2_O_2_ in CH_2_Cl_2_.

Solutions of complexes [Mo(PNP^Me^‐*i*Pr)(O)X]^+^ (**3a**,**b**) in chlorinated solvents such as CHCl_3_ and CH_2_Cl_2_ are air sensitive being slowly oxidized to yield the mono oxo Mo^VI^ complex [Mo(κ^2^ *O,O*‐ONO^Me^‐*i*Pr)(O)Cl_3_]SbF_6_ (**5**) (Scheme 4). The same reaction takes place rapidly in the presence of H_2_O_2_ yielding quantitatively complex **5** within 10 minutes as monitored by ^31^P{^1^H} NMR spectroscopy. During this reaction, three chloride ligands from the solvent replace both iodo and bromo ligands, while the phosphine moieties are oxidized to the respective phosphine oxides. The pyridine ring is no longer coordinated, while the phosphine oxide moieties are coordinated via the oxygen atoms. In this context, it has to be noted that if the solvent is CH_2_Cl_2_ instead of acetone, [Mo(PNP^Me^‐*i*Pr)(CO)(X)(solv)]^+^ (**2a**,**b**) reacts with air and an excess of water to afford the cationic mono oxo complexes [Mo(PNP^Me^‐*i*Pr)(O)X]^+^ (**3a**,**b**), but also small amounts of the Mo^VI^ species **5** (ca 15 %). Although we could not directly detect H_2_O_2_, this observation suggests that during this reaction H_2_O_2_ may be released (vide infra) as this oxidation process is very slow in the presence of oxygen, but fast in the presence of H_2_O_2_. Moreover, H_2_O_2_ could disproportionate under these reaction conditions to form water and O_2_ which again would form **3a**,**b** from complexes **2a**,**b**.^20^ Complex **5** is isolated in essentially quantitative yield and was characterized by elemental analysis, ^1^H, ^13^C{^1^H}, and ^31^P{^1^H} NMR spectroscopy. In addition, **5** was characterized by X‐ray crystallography.

**Figure 4:**
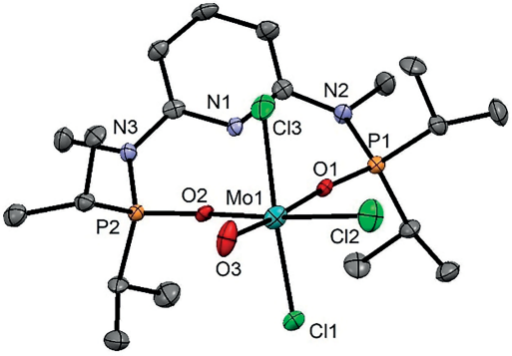
Structural diagram of [Mo(κ^2^ *O,O*‐ONO^Me^‐*i*Pr)(O)Cl_3_]SbF_6_ **·**1/2CH_2_Cl_2_ (**5·**1/2CH_2_Cl_2_) showing displacement ellipsoids at the 50 % probability level (hydrogen atoms, solvent, and SbF_6_^–^ counterion omitted for clarity). Mo1–Cl1 2.371(2), Mo1–Cl2 2.297(2), Mo1–Cl3 2.368(2), Mo1–O1 2.212(5), Mo1–O2 2.077(4), Mo1–O3 1.719(6), Cl1–Mo1–Cl2 91.43(8), Cl1–Mo1–Cl3 166.71(8), Cl1–Mo1–O1 84.7(1), O1–Mo1–O2 76.1(2), O1–Mo1–O3 171.5(2), O2–Mo1–O3 95.5(2).

A structural view of **5** is depicted in Figure 4 with selected bond lengths and angles given in the caption. This complex adopts an octahedral geometry with the oxygen atoms of the oxidized PNP ligand being coordinated in *cis*‐κ^2^ *O,O*‐fashion. The Mo1–O3 distance is 1.719(6) Å which is typical for a Mo=O double bond and thus significantly longer than the Mo–O bond in **3a** [1.663(2) Å].

**Figure 5:**
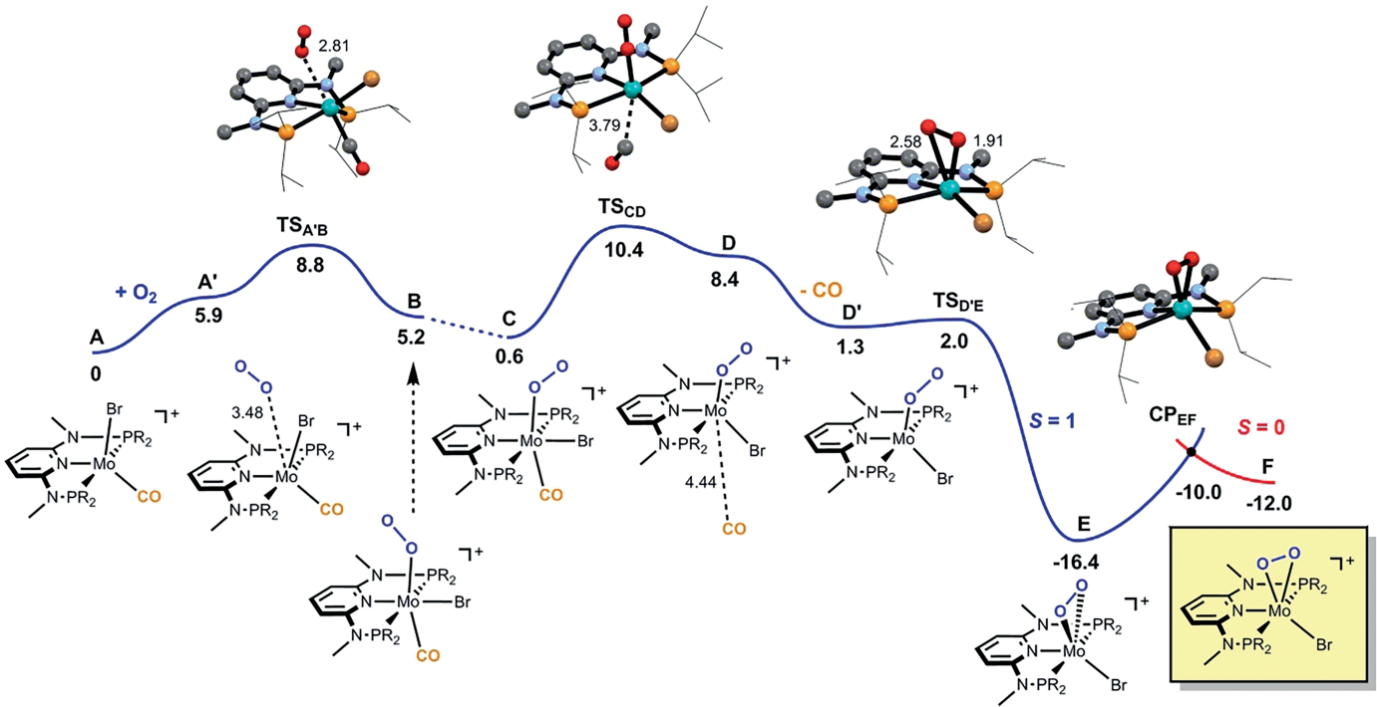
Free energy profile calculated for the oxidation of complex **A**. The free energy values [kcal/mol] are referred to the initial reactants (**A** + O_2_) and relevant distances [Å] are presented.

**Figure 6:**
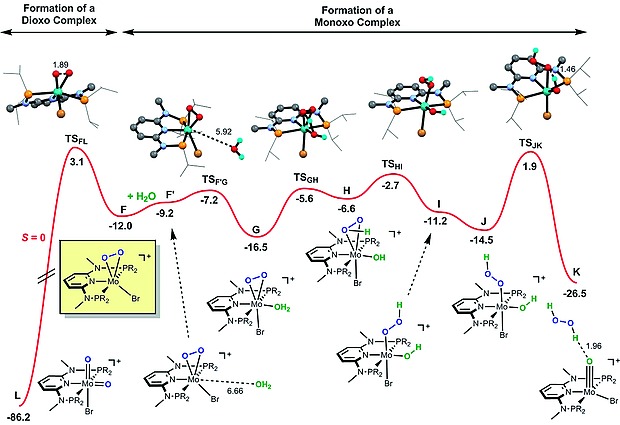
Free energy profile calculated for the competitive formation of mono‐ and dioxo complexes **K** and **L**, respectively. The free energy values [kcal/mol] are referred to the initial reactants (**A** + O_2_) and relevant distances [Å] are presented.

One possible, but reasonable mechanism, accounting for the role of dioxygen and water was established by means of DFT calculations. Free energy profiles are represented in Figures 5 and 6. Intermediate **2b** (**A** in the profile), formed after Br^–^ removal from **1b**, readily coordinates O_2_. The reaction proceeds along the spin triplet Potential Energy Surface (PES) starting with **A′**, the pair of reactants of **2b** and O_2_ (O_2_ being a triplet) producing complex **B** via transition state **TS_A′B_**. In this transition state the new Mo–O bond is only incipient with a distance of 2.81 Å which is still far away from the coordination distance of 2.05 Å in **B**. The energy barrier is 8.8 kcal/mol. After re‐orientation of the O_2_ ligand to afford **C** the process is practically thermoneutral with respect to the initial reagents (**C** is only 0.6 kcal/mol less stable than the separated reactants).

The next step involves CO dissociation from **C**. This step has a barrier of 9.8 kcal/mol (**TS_CD_**) and yields a coordinatively unsaturated species with an O_2_ ligand and the halide, beside the PNP ligand (**D** and **D′**). The transition state **TS_CD_** is a late one with a Mo–C(CO) separation of 3.79 Å. The entire process from **C** to **D′** is essentially thermoneutral (Δ*G* = 0.7 kcal/mol). Dissociation of the CO ligand directly from **2b** is unfavorable with Δ*G* = 34 kcal/mol and thus coordination of dioxygen is required. From **D′** there is coordination of the dangling O‐atom with formation of a peroxide κ^2^‐O_2_ ligand corresponding to an oxidative addition with the metal changing from Mo^II^ in **D′** to Mo^IV^ in **E**. This is a facile process with a barrier of only 0.7 kcal/mol (**TS_D′E_**). In the transition state, the new Mo–O bond is about to be formed with a distance of 2.58 Å. This is significantly longer than the Mo–O bonds in **E** (1.97 Å). Formation of the peroxide complex is thermodynamically favorable with **E** being 16.4 kcal/mol more stable than the initial reagents. The reaction then proceeds from **E** to **F** with a change in spin state from triplet (*S* = 1) to singlet (*S* = 0). That corresponds to a “spin‐forbidden” or “non‐adiabatic” reaction and, thus, its profile goes through a minimum‐energy crossing point (MECP) of the two potential energy surfaces (PES) involved.^21^, ^22^ The barrier calculated for the spin change of **E** is 6.4 kcal/mol (**CP_EF_**) but the spin singlet intermediate **F** is 4.4 kcal/mol less stable than its high spin counterpart, and, thus corresponds to a rather facile but noticeably endergonic step.

Following intermediate **F**, the reaction profile proceeds along the spin singlet PES (Figure 6). There are two alternative paths. In one case, there is O–O bond cleavage via an oxidative addition process that leads to the di‐oxo Mo^VI^ complex **L**. This is a single‐step process represented on the left side of the profile in Figure 6, being highly exergonic as the product **L** is 86.2 kcal/mol more stable than the initial reagents. The barrier associated with this step is 15.1 kcal/mol. In the corresponding transition state **TS_FL_**, the O–O is already clearly elongated (*d*_O–O_ = 1.89 Å) when compared to the distance in intermediate **F** (*d*_O–O_ = 1.47 Å). In agreement with these calculations, it was experimentally shown that the oxo ligands in this complex indeed stem from dioxygen.

In the presence of water, **F** reacts readily to form the water adduct **G**. This reaction is facile with a barrier of only 4.8 kcal/mol. The corresponding transition state **TS_F′G_** is an early one with the incoming water molecule quite remote from the metal center (*d*_Mo–O_ = 5.92 Å) and the process is exergonic by 7.3 kcal/mol. Intermediate **G**, adopts a pseudo‐octahedral coordination around the metal with the O_2_ and the halide in opposite positions, and the water *trans* to pyridine N‐atom. From **G** to **H** there is H‐transfer from the water to the O_2_ ligand, transforming the peroxide into a hydroperoxide κ^2^‐HOO ligand. This process has a barrier of 10.9 kcal/mol and is clearly endergonic (Δ*G* = 9.9 kcal/mol). In transition state **TS_GH_** the new O–H bond is almost formed (1.14 Å) while the former one, H–O(water) is practically broken (1.32 Å). From **H** to **I** the hydroperoxide goes from κ^2^‐ to κ^1^‐coordination with the breaking of one Mo–O bond in a facile process with a barrier of 3.9 kcal/mol. In the corresponding transition state **TS_HI_** the Mo–O distance (2.68 Å) is already 0.46 Å longer than the one existing in the κ^2^‐HOO intermediate **H**. From **I** to **J** there is a re‐orientation of the κ^1^‐HOO ligand and, then in a final step takes place H‐transfer from the hydroxo ligand to the second O‐atom in HCOO, forming hydrogen peroxide and the mono‐oxo complex [Mo(PNP^Me^‐*i*Pr)(O)(Br)]^+^ in **K**. In transition state **TS_JK_** the new O–H bond is still far from formed (*d* = 1.46 Å) and the H–O(OH) bond is practically intact (1.07 Å), only 0.1 Å longer than the one present in intermediate **J**. Also, in **TS_JK_** the hydroperoxide ligand is starting to dissociate and the Mo–O distance is 0.33 Å longer than in **J**. In this process one H_2_O_2_ molecule will be released. This last step has a barrier of 16.4 kcal/mol and is clearly exergonic with Δ*G* = –12.0 kcal/mol, resulting in a final product 26.5 kcal/mol more stable than **A**. In the formation of the mono‐oxo complex (from **F** to **K**) the least stable transition state is **TS_JK_** with a free energy 1.9 kcal/mol above the initial reactants. On the other hand, transition state **TS_FL_** associated with the formation of the di‐oxo product (from **F** to **L**) has an energy of 3.1 kcal/mol relative to **A**. The difference between the total barriers of the two paths is only 1.2 kcal/mol and, thus, they can be considered competitive. The formation of the mono‐oxo complex [Mo(PNP^Me^‐*i*Pr)(O)(Br)]^+^ (**K**), following water addition to the intermediate with a κ^2^‐peroxide ligand (**F**), is slightly more favorable than O–O splitting with formation of the corresponding di‐oxo species [Mo(PNP^Me^‐*i*Pr)(O)_2_(Br)]^+^ (**L**). However, in the presence of excess of H_2_O this pathway may become the predominating one. Importantly, in the calculated mechanism the O‐ligand in the final complex is originated from the incoming water molecule, in agreement with the experimental results obtain with H_2_^18^O.

## Conclusions

In sum, we have prepared and fully characterized new cationic mono oxo Mo^IV^ PNP pincer complexes of the type [Mo(PNP^Me^‐*i*Pr)(O)X]^+^ (X = I, Br). These compounds are coordinatively unsaturated and feature a strong Mo–O triple bond. This bonding mode is supported by DFT calculations. ESI‐MS measurements with ^18^O labeled water (H_2_^18^O) and molecular oxygen (^18^O_2_) reveal that the formation of these complexes requires an interplay between water and molecular oxygen. The major source of oxygen of the Mo≡O oxo bond appears to be water. The crystal structure of [Mo(PNP^Me^‐*i*Pr)(O)I]SbF_6_ is presented. Detailed theoretical studies based on DFT calculations established a reasonable mechanism for the formation of both mono and dioxo molybdenum complexes proceeding via two competitive pathways.

## Experimental Section

**General**

All manipulations were performed under an inert atmosphere of argon by using Schlenk techniques. The solvents were purified according to standard procedures.^23^ H_2_^18^O (97.0 % ^18^O), ^18^O_2_ (97.0 % ^18^O) and all deuterated solvents were purchased from Sigma–Aldrich and used without further purification. The deuterated solvents were dried with 4 Å molecular sieves. Complexes [Mo(PNP^Me^‐*i*Pr)(CO)I_2_] (**1a**) and [Mo(PNP^Me^‐*i*Pr)(CO)Br_2_] (**1b**) were prepared according to the literature.^12^ ^1^H, ^13^C{^1^H}, and ^31^P{^1^H} NMR spectra were recorded on Bruker AVANCE‐250, AVANCE‐300 DPX, and AVANCE‐400 spectrometers. ^1^H and ^13^C{^1^H} NMR spectra were referenced internally to residual protio‐solvent and solvent resonances, respectively, and are reported relative to tetramethylsilane (*δ* = 0 ppm). ^31^P{^1^H} NMR spectra were referenced externally to H_3_PO_4_ (85 %) (*δ* = 0 ppm).

Mass spectrometric measurements were performed on an Esquire 3000^*plus*^ 3D‐quadrupole ion trap mass spectrometer (Bruker Daltonics, Bremen, Germany) in positive‐ion mode electrospray ionization (ESI‐MS). Mass calibration was done with a commercial mixture of perfluorinated trialkyltriazines (ES Tuning Mix, Agilent Technologies, Santa Clara, CA, USA). All analytes were dissolved in CH_3_CN “Lichrosolv” quality (Merck, Darmstadt, Germany) to a concentration of roughly 1 mg/mL and doped with sodium halides (Merck, Darmstadt, Germany) to avoid or suppress dissociation of halogen substituents from the complexes. Direct infusion experiments were carried out using a Cole Parmer model 74900 syringe pump (Cole Parmer Instruments, Vernon Hills, IL, USA) at a flow rate of 2 µL/min. Full scan and MS/MS‐scans were measured in the range *m/z* 100–1000 with the target mass set to *m/z* 800. Further experimental conditions include: drying gas temperature: 150 °C; capillary voltage: –4 kV; skimmer voltage: 40 V; octapole and lens voltages: according to the target mass set. Helium was used as buffer gas for full scans and as collision gas for MS/MS‐scans in the low energy collision induced dissociation (CID) mode. The activation and fragmentation width for tandem mass spectrometric (MS/MS) experiments was set to 10–12 Da to cover the entire isotope cluster for fragmentation. The corresponding fragmentation amplitude ranged from 0.3 to 0.8 V to keep a low abundant precursor ion intensity in the resulting MS/MS spectrum. All mass calculations are based on the lowest mass isotope for molybdenum (^92^Mo‐isotope). Mass spectra and tandem spectra were averaged during data acquisition time of 1 to 2 min and one analytical scan consisted of five successive micro scans resulting in 50 and 100 analytical scans, respectively, for the final mass spectrum or MS/MS spectrum.

The labelling experiments were performed on a LTQ‐XL linear ion trap mass spectrometer (Thermo Fisher Scientific) fitted with an electrospray ionization (ESI) source operating in the positive ion mode.

**Instrumental Analysis Conditions:** [Mo(PNP^Me^‐*i*Pr)(CO)I_2_] (**1a**) was dissolved in acetonitrile to the millimolar concentration and doped with sodium iodide. Sample solutions were infused at a flow rate of 3–5 µL/min via the instrument′s on‐board syringe pump directly connected to the ESI source. Typical experimental conditions were: source voltage 4–5 kV, capillary temperature 200 °C. Nitrogen was used as sheath and auxiliary gas at a flow rate of 15 and 5 arbitrary units (a.u. ≈ 0.37 L/min). Full scan mass spectra were measured in the *m/z* range 100–1000 and were the average of 25–50 scans, each resulting from three micro scans. Two sets of separate and different labelling experiments, using either ^18^O_2_ or H_2_^18^O were performed, as described in the following.

**Experiment 1 with ^18^O_2_:** A flask containing a mixture of [Mo(PNP^Me^‐*i*Pr)(CO)I_2_] (**1a**)/NaI was connected to a vacuum system and carefully evacuated. It was then filled with ^18^O_2_ (760 Torr) and acetonitrile was subsequently added to the solid mixture using a gas tight syringe to avoid contact with air. The solution was stirred and the flask was left at ambient temperature. Samples taken at different period of times (15 min, 1 h, 3 h, 20 h) were infused into the ESI source and analyzed using instrumental conditions as described in the paragraph above. As the ESI source is an atmospheric pressure ionization (API) source, the contact with (moist) air cannot be avoided. ^16^O sources can thus come from: (1) residual ^16^O_2_ present as impurity in the labelled sample; (2) residual O_2_ possibly left in the flask after evacuation from the air and H_2_O adsorbed in the glass walls of the flask or present in the acetonitrile solvent; (3) O_2_ and H_2_O from the air, always present in such mass spectrometers.

**Experiment 2 with H_2_^18^O:** A sample of [Mo(PNP^Me^‐*i*Pr)(CO)I_2_] (**1a**)/NaI was introduced in a sealed vial and dissolved in a mixture of acetonitrile/H_2_^18^O (15 % H_2_^18^O v/v) injected through the rubber septum capping the vial. Samples were promptly taken and infused into the ESI source, using experimental parameters as described above. As in the previous experiments with ^18^O_2_, sources of ^16^O are present, the major ones coming from: (1) residual H_2_^16^O is present as impurity in the labelled sample and is possibly present in acetonitrile; (2) O_2_ and H_2_O from air are always present in this type of mass spectrometers. The ion–molecule reactions were performed on the LTQ XL linear ion trap mass exploiting an in‐house modification that allows the introduction of neutral gases into the ion trap in order to observe ion–molecule reactions of mass‐selected ions with the neutral reagent (O_2_ and H_2_O), as described in details elsewhere.^24^ Ionic species generated in the electrospray source were isolated with an isolation width of 1 *m/z* and reacted with the neutral of interest for different periods of time. For each reaction time, mass spectra were recorded using an injection time of 200 ms, a normalized collision energy set to zero, and the activation Q value optimized to ensure stable trapping fields for all ions. Spectra were acquired using the MS^n^ function of the Xcalibur 2.0.6 software to mass‐select the precursor ion. All the spectra are the average of 10 scans for each acquisition.

**Reaction of [Mo(PNP^Me^‐*i*Pr)(CO)I_2_] (1a) with AgSbF_6_ in [D_6_]Acetone:** A solution of [Mo(PNP^Me^‐*i*Pr)(CO)I_2_] (**1a**) (50 mg, 0.065 mmol) in [D_6_]acetone was treated with AgSbF_6_ (0.065 mmol). The reaction was followed by ^31^P{^1^H} NMR and IR showing he quantitative formation of complex [MoPNP^Me^‐*i*Pr(CO)I]^+^ (**2a**). ^31^P{^1^H} NMR ([D_6_]acetone, 20 °C): *δ* = 183.3 ppm. IR (ATR): ν̃ = 1832 (ν_CO_). After the solution is exposed to air and treated with water [Mo(PNP^Me^‐*i*Pr)(O)I]^+^ (**3a**) is formed together with small amounts of [Mo(PNP^Me^‐*i*Pr)(CO)_3_I]^+^ (*δ* = 137.1 ppm. in the ^31^P{^1^H} NMR spectrum).

**Reaction of [Mo(PNP^Me^‐*i*Pr)(CO)Br_2_] (1b) with AgSbF_6_ in [D_6_]Acetone:** A solution of [Mo(PNP^Me^‐*i*Pr)(CO)Br_2_] (**1b**) (50 mg, 0.075 mmol) in [D_6_]acetone was treated with AgSbF_6_ (0.075 mmol). The reaction was followed by ^31^P{^1^H} NMR and IR showing the quantitative formation of complex [Mo(PNP^Me^‐*i*Pr)(CO)Br]^+^ (**2b**). ^31^P{^1^H} NMR ([D_6_]acetone, 20 °C): *δ* = 189.5 ppm. IR (ATR): ν̃ = 1840 (ν_CO_). After the solution is exposed to air and treated with water [Mo(PNP^Me^‐*i*Pr)(O)Br]^+^ (**3a**) is formed together with small amounts of [Mo(PNP^Me^‐*i*Pr)(CO)_3_Br]^+^ (*δ* = 132.0 ppm. in the ^31^P{^1^H} NMR spectrum).

**[Mo(PNP^Me^‐*i*Pr)(O)I]SbF_6_ (3a):** A solution of [Mo(PNP^Me^‐*i*Pr)(CO)I_2_] (**1a**) (100 mg, 0.13 mmol) in acetone (10 mL) was treated with AgSbF_6_ (45.98 mg, 0.13 mmol) and the mixture was stirred for 4 h. After filtration through glass wool and Celite, the solution was exposed to air for 2 min and an excess of H_2_O (3 mL, 0.17 mol) was added. After 30 min the solution was filtered over glass wool and Celite and the solvent was removed under reduced pressure. A green solid was obtained which was washed twice with *n*‐pentane and then dried under vacuum. Yield: 58.7 mg (72 %). C_19_H_37_F_6_IMoN_3_OP_2_Sb (844.09): calcd. C 27.04, H 4.42, N 4.98; found C 27.20, H 4.46, N 5.04. ^1^H NMR (CD_2_Cl_2_, 20 °C): *δ* = 8.05 (tt, *J* = 8.5, *J* = 1.2 Hz, 1 H, py^4^), 6.60 (d, *J* = 8.5 Hz, 2 H, py^3,5^), 3.49–3.33 (m, 2 H, CH), 3.33–3.28 (m, 6 H, NCH_3_), 3.16–2.94 (m, 2 H, CH), 1.71–1.53 (m, 6 H, CH_3_), 1.50–136 (m, 6 H, CH_3_), 1.31–1.17 (m, 6 H, CH_3_), 0.79–0.63 (m, 6 H, CH_3_) ppm. ^13^C{^1^H} NMR (CD_2_Cl_2_, 20 °C): *δ* = 165.3 (vt, *J* = 7.4 Hz, py^2,6^), 148.1 (py^4^), 100.9 (vt, *J* = 3.0 Hz, py^3,5^), 36.0 (NCH_3_), 28.2 (t, *J* = 11.6 Hz, CH), 23.5 (t, *J* = 9.9 Hz, CH), 18.0 (vt, *J* = 5.4 Hz, CH_3_), 17.4 (vt, *J* = 2.3 Hz, CH_3_), 17.1 (CH_3_), 16.6 (CH_3_) ppm. ^31^P{^1^H} NMR (CD_2_Cl_2_, 20 °C): *δ* = 149.2 ppm. IR (ATR): ν̃ = 955 (ν_M=O_). ESI MS (CH_3_CN) positive ion: *m/z* = [M]^+^ 604.1.

**[Mo(PNP^Me^‐*i*Pr)(O)Br]SbF_6_ (3b):** This complex was prepared analogously to **3a** with [Mo(PNP^Me^‐*i*Pr)(CO)Br_2_] (**1b**) (100 mg, 0.15 mmol) and AgSbF_6_ (52.6 mg, 0.15 mmol) as starting materials. Yield: 56.7 mg (66 %). C_19_H_37_BrF_6_MoN_3_OP_2_Sb (797.09): calcd. C 28.63, H 4.68, N 5.27; found C 28.56, H 4.70, N 5.40. ^1^H: *δ* = NMR (CD_2_Cl_2_, 20 °C): 8.06 (t, *J* = 8.2 Hz, 1 H, py^4^), 6.63 (d, *J* = 8.3 Hz, 2 H, py^3,5^), 3.32 (s, 6 H, NCH_3_), 2.88–2.74 (m, 2 H, CH), 2.68–2.56 (m, 4 H, CH), 1.76–1.46 (m, 12 H, CH_3_), 1.44–0.96 (m, 12 H, CH_3_) ppm. ^13^C{^1^H} NMR (CD_2_Cl_2_, 20 °C): *δ* = 161.9 (vt, *J* = 7.0 Hz, py^2,6^), 146.6 (py^4^), 101.7 (vt, *J* = 2.6 Hz, py^3,5^), 36.6 (NCH_3_), 31.6 (t, *J* = 12.8 Hz, CH), 29.8 (t, *J* = 8.6 Hz, CH), 20.2 (CH_3_), 19.4 (br., CH_3_), 18.6 (vt, *J* = 5.8 Hz, CH_3_), 17.2 (CH_3_) ppm. ^31^P{^1^H}: *δ* = NMR (CD_2_Cl_2_, 20 °C): 145.2 ppm. IR (ATR): ν̃ = 940 (ν_M=O_). ESI MS (CH_3_CN) positive ion: *m/z* = [M]^+^ 555.9.

**[Mo(κ^2^*O*,*O*‐ONO^Me^‐*i*Pr)(O)Cl_3_]SbF_6_·(5)**

**Method A:** A solution of **3a** (50 mg, 0.082 mmol) in CH_2_Cl_2_ or CHCl_3_ (10 mL) was exposed to air for 4 d at room temperature. After that, the solution was filtered and the solvent was removed. The product was obtained as a red solid which was washed twice with *n*‐pentane and then dried under vacuum. Yield: 46.87 mg (92 %). C_19_H_37_Cl_3_F_6_MoN_3_O_3_P_2_Sb (855.53): calcd. C 26.67, H 4.36, N 4.91; found C 26.73, H 4.35, N 5.10. ^1^H NMR (CD_2_Cl_2_, 20 °C): *δ* = 7.95 (t, *J *= 8.6 Hz, 1 H, py^4^), 7.02 (d, *J *= 8.5 Hz, 2 H, py^3,5^), 3.10 (d, *J* = 7.2 Hz, 6 H, NCH_3_), 2.59–2.37 (m, 4 H, CH), 1.38–1.15 (m, 24 H, CH_3_) ppm. ^13^C{^1^H} NMR (CD_2_Cl_2_, 20 °C): *δ* = 153.0 (py^2,6^), 145.7 (py^4^), 105.1 (py^3,5^), 33.6 (NCH_3_), 27.4 (CH), 26.2 (CH), 16.3 (CH_3_), 15.5 (d, *J* = 4.2 Hz, CH_3_), 15.2 (CH_3_) ppm. ^31^P{^1^H} NMR (CD_2_Cl_2_, 20 °C): *δ* = 73.7 ppm. The same reaction takes place with **3b** affording **5** in 87 % yield.

**Method B:** A solution of [Mo(PNP^Me^‐*i*Pr)(O)I]^+^ (**3a**) (50 mg, 0.082 mmol) in CH_2_Cl_2_ (10 mL) was treated with 3.5 equiv. H_2_O_2_ (30 % in H_2_O, 29 µL, 0.288 mmol) and the mixture was stirred for 30 min. After that, the solution was filtered and the solvent was removed. The product was obtained as a red solid which was washed twice with *n*‐pentane and then dried under vacuum. Yield: 49.9 mg (98 %).

**Method C:** A solution of [Mo(PNP^Me^‐*i*Pr)(CO)I]^+^ (**2a**) (50 mg, 0.081 mmol) in CH_2_Cl_2_ (10 mL) was treated with H_2_O_2_ (30 % in H_2_O, 29 µL, 0.288 mmol) and the mixture was stirred for 2 h. After that, the solution was filtered and the solvent was removed. The product was obtained as a red solid which was washed twice with *n*‐pentane and then dried under vacuum. Yield: 48.0 mg (96 %).

**Reaction of [Mo(PNP^Me^‐*i*Pr)(O)I]^+^ (3a) with H_2_O_2_ in CD_2_Cl_2_:** A solution of [Mo(PNP^Me^‐*i*Pr)(O)I]^+^ (**3a**) (20 mg, 0.033 mmol) in CD_2_Cl_2_ (0.7 mL) was treated with H_2_O_2_ (30 % in H_2_O, 15 µL, 0.144 mmol). The reaction was followed by ^31^P{^1^H} NMR and quantitative formation of complex [Mo(κ^2^ *O,O*‐ONO^Me^‐*i*Pr)(O)Cl_3_]SbF_6_ **·**(**5**) was observed after 10 min.

**Crystal Structure Determination**

Single crystals of **3a** and **5·**1/2CH_2_Cl_2_ were pre‐selected, embedded in perfluorinated polyether and mounted on Kapton micro mounts. X‐ray diffraction data were measured in a cold stream of nitrogen at *T* = 100 K on a Bruker APEX‐II diffractometer^25^ with Mo‐*K*_α_ radiation. After integration of the data with the program SAINT,^25^ an absorption correction based on the semi‐empirical “multi‐scan” approach was performed with the SADABS program.^25^ The crystal structures were solved using the dual space approach implemented in SHELXT^26^ and was refined using the SHELXL program package.^26^ All H atoms were placed geometrically and refined in the riding model approximation, with C‐H = 1.00 Å and *U*_iso_(H) = 1.2*U*_eq_(C) for the CH groups and with C‐H = 0.98 Å and *U*_iso_(H) = 1.5*U*_eq_(C) for the methyl groups. All non‐hydrogen atoms were refined anisotropically. Molecular graphics were generated with the program MERCURY.^27^

https://www.ccdc.cam.ac.uk/services/structures?id=doi:10.1002/ejic.201701413 1480834 (for **3a**) and 1574491 (for **5·**1/2CH_2_Cl_2_) contain the supplementary crystallographic data for this paper. These data can be obtained free of charge from http://www.ccdc.cam.ac.uk/.

**Computational Details**

The computational results presented have been achieved in part using the Vienna Scientific Cluster (VSC). Calculations were performed using the Gaussian 09 software package,^28^ and the B3LYP functional, without symmetry constraints. That functional include a mixture of Hartree–Fock^29^ exchange with DFT exchange‐correlation, given by Becke's three parameter functional^30^ with the Lee, Yang and Parr correlation functional, which includes both local and non‐local terms.^31^, ^32^ The optimized geometries were obtained with the Stuttgart/Dresden ECP (SDD) basis set^33^ to describe the electrons of Mo and I, and a standard 6‐31G** basis set^34^ for the other atoms. Transition state optimizations were performed with the Synchronous Transit‐Guided Quasi‐Newton Method (STQN) developed by Schlegel et al.,^35^ following extensive searches of the Potential Energy Surface. Frequency calculations were performed to confirm the nature of the stationary points, yielding one imaginary frequency for the transition states and none for the minima. Each transition state was further confirmed by following its vibrational mode downhill on both sides, and obtaining the minima presented on the energy profiles. The electronic energies were converted to free energy at 298.15 K and 1 atm by using zero‐point energy and thermal energy corrections based on structural and vibration frequency data calculated at the same level.

The Minimum Energy Crossing Point (MECP) between the spin singlet (*S* = 0) and the spin triplet (*S* = 1) Potential Energy Surfaces (PES) was determined using a code developed by Harvey et al.^36^ This code consists of a set of shell scripts and Fortran programs that uses the Gaussian results of energies and gradients of both spin states to produce an effective gradient pointing towards the MECP. This is not a stationary point and, hence, a standard frequency analysis is not applicable. Therefore, the free energy value of the crossing point (**CP_EF_**) was obtained through frequency calculations projected for vibrations perpendicular to the reaction path.^37^ Orbital representations were obtained with Molekel.^38^

**Supporting Information** (see footnote on the first page of this article): Atomic coordinates of all optimized species (*xyz* files).

## Supporting information

Supporting Information
