# Supplementary material for: Formation of Mono Oxo Molybdenum(IV) PNP Pincer Complexes: Interplay between Water and Molecular Oxygen
Source: Eur J Inorg Chem. 2018 Feb 12;2018(7):876–84. doi: 10.1002/ejic.201701413 (PMC6485545; doi:10.1002/ejic.201701413)
Supplement: Supplementary file 1 — Supporting Information [file EJIC-2018-876-s001.pdf]

*Eur. J. Inorg. Chem.* · ISSN 1099–0682

https://doi.org/10.1002/ejic.201701413

# SUPPORTING INFORMATION

**Title:** Formation of Mono Oxo Molybdenum(IV) PNP Pincer Complexes: Interplay between Water and Molecular Oxygen

**Author(s):** Sara R. M. M. de Aguiar, Özgür Öztopcu, Anna Troiani, Giulia de Petris, Matthias Weil, Berthold Stöger, Ernst Pittenauer, Günter Allmaier, Luis F. Veiros, Karl Kirchner*

65
Complex A

| | | | |
|---|---|---|---|
| P | 2.109240 | 0.338470 | -0.010589 |
| P | -2.259738 | -0.116114 | 0.027900 |
| N | -0.334638 | 1.772846 | -0.502016 |
| N | 1.927057 | 2.077820 | 0.061041 |
| N | -2.622024 | 1.389723 | -0.797799 |
| C | 0.720619 | 2.628941 | -0.327175 |
| C | 0.576856 | 4.012067 | -0.523126 |
| C | -0.673637 | 4.514443 | -0.852831 |
| C | -1.772739 | 3.670709 | -0.959507 |
| C | -1.578052 | 2.300087 | -0.762366 |
| C | 2.836744 | -0.073388 | 1.667219 |
| C | 1.841139 | 0.280640 | 2.783903 |
| C | 3.440561 | 0.039164 | -1.325333 |
| C | 4.862472 | 0.493406 | -0.948513 |
| C | -3.504556 | 1.279217 | 2.228096 |
| C | -2.427479 | -0.971125 | 2.756080 |
| C | -3.596871 | -1.319976 | -0.512465 |
| C | -4.981953 | -1.130659 | 0.138810 |
| C | 0.105974 | -1.801483 | 0.404801 |
| O | 0.164591 | -2.675877 | 1.187720 |
| H | 1.423421 | 4.675538 | -0.428033 |
| H | -0.798449 | 5.581619 | -1.007339 |
| H | -2.757696 | 4.068429 | -1.156378 |
| H | 2.316257 | 0.117992 | 3.756515 |
| H | 0.953384 | -0.355855 | 2.736203 |
| H | 1.523280 | 1.326678 | 2.739997 |
| H | 5.525066 | 0.304038 | -1.799270 |
| H | 5.261865 | -0.068198 | -0.099293 |
| H | 4.928223 | 1.558595 | -0.722548 |
| H | -3.409166 | 1.541234 | 3.287157 |
| H | -4.502045 | 0.856253 | 2.088101 |
| H | -3.435536 | 2.208268 | 1.656445 |
| H | -2.317953 | -0.662041 | 3.800762 |
| H | -1.625545 | -1.677063 | 2.534459 |
| H | -3.381722 | -1.497723 | 2.675951 |
| H | -5.695938 | -1.779362 | -0.378731 |
| H | -5.367343 | -0.111326 | 0.085344 |
| H | -4.974165 | -1.433733 | 1.188149 |
| C | -3.128921 | -2.780205 | -0.340526 |
| H | -2.220599 | -2.991559 | -0.904813 |
| H | -2.957472 | -3.036039 | 0.708135 |
| H | -3.917733 | -3.439865 | -0.715317 |
| C | 3.451806 | -1.419827 | -1.824843 |
| H | 2.469697 | -1.763206 | -2.156242 |
| H | 4.130196 | -1.491522 | -2.680917 |
| H | 3.812747 | -2.113545 | -1.061602 |
| C | 3.326563 | -1.528842 | 1.779959 |
| H | 4.123531 | -1.758088 | 1.069871 |
| H | 3.735568 | -1.680085 | 2.784150 |
| H | 2.518946 | -2.249425 | 1.642905 |
| H | -1.425545 | 0.783116 | 2.034254 |
| C | -2.388113 | 0.284979 | 1.864999 |
| H | -3.671579 | -1.122328 | -1.589736 |
| H | 3.083871 | 0.673418 | -2.148819 |
| H | 3.715032 | 0.575819 | 1.768958 |
| Mo | -0.037400 | -0.433920 | -0.914439 |
| Br | -0.390424 | -1.805816 | -2.925093 |
| C | 2.997092 | 2.998075 | 0.474665 |

| | | | |
|---|---|---|---|
| H | 2.616414 | 3.714703 | 1.208267 |
| H | 3.802581 | 2.439295 | 0.944221 |
| H | 3.412610 | 3.546547 | -0.377780 |
| C | -3.934144 | 1.832975 | -1.280715 |
| H | -3.803224 | 2.410097 | -2.199337 |
| H | -4.544506 | 0.967907 | -1.527214 |
| H | -4.470355 | 2.445471 | -0.545857 |

67

Complex A’

| | | | |
|---|---|---|---|
| Mo | -1.049263 | -1.117873 | -1.334235 |
| P | 1.103094 | -0.329249 | -0.453166 |
| P | -3.270280 | -0.794279 | -0.386855 |
| N | -1.353032 | 1.093720 | -0.915965 |
| N | 0.902949 | 1.405024 | -0.338272 |
| N | -3.634368 | 0.699642 | -1.233184 |
| C | -0.304472 | 1.953320 | -0.727725 |
| C | -0.453876 | 3.337615 | -0.912778 |
| C | -1.703184 | 3.836020 | -1.252172 |
| C | -2.796337 | 2.986742 | -1.377109 |
| C | -2.595479 | 1.616315 | -1.185966 |
| C | 1.861294 | -0.776912 | 1.201058 |
| C | 0.887251 | -0.456219 | 2.346439 |
| C | 2.421204 | -0.577352 | -1.791447 |
| C | 3.846006 | -0.135817 | -1.411280 |
| C | -4.498528 | 0.639413 | 1.797433 |
| C | -3.425751 | -1.604707 | 2.356936 |
| C | -4.612409 | -2.004574 | -0.897784 |
| C | -5.997057 | -1.789550 | -0.253556 |
| C | -0.900830 | -2.465615 | 0.001686 |
| O | -0.839713 | -3.329383 | 0.796088 |
| H | 0.388533 | 4.004479 | -0.804965 |
| H | -1.832128 | 4.903650 | -1.400144 |
| H | -3.781784 | 3.379803 | -1.581169 |
| H | 1.384715 | -0.637191 | 3.304562 |
| H | 0.003593 | -1.098455 | 2.303842 |
| H | 0.561061 | 0.588044 | 2.333250 |
| H | 4.499071 | -0.280977 | -2.278012 |
| H | 4.257889 | -0.735718 | -0.594848 |
| H | 3.910377 | 0.917469 | -1.134086 |
| H | -4.395843 | 0.919969 | 2.851068 |
| H | -5.497750 | 0.216150 | 1.671416 |
| H | -4.431509 | 1.558166 | 1.209083 |
| H | -3.306725 | -1.278812 | 3.395460 |
| H | -2.629058 | -2.318340 | 2.141099 |
| H | -4.383425 | -2.127191 | 2.292729 |
| H | -6.714629 | -2.446418 | -0.755669 |
| H | -6.375673 | -0.769439 | -0.333067 |
| H | -5.992318 | -2.066753 | 0.803002 |
| C | -4.153580 | -3.462463 | -0.685094 |
| H | -3.244715 | -3.694974 | -1.239871 |
| H | -3.988345 | -3.691229 | 0.370685 |
| H | -4.945229 | -4.126830 | -1.045364 |
| C | 2.427583 | -2.015663 | -2.347051 |
| H | 1.442482 | -2.346898 | -2.682873 |
| H | 3.099059 | -2.056082 | -3.210741 |
| H | 2.793362 | -2.738783 | -1.613850 |
| C | 2.357915 | -2.233347 | 1.264284 |
| H | 3.142183 | -2.439799 | 0.533241 |
| H | 2.786148 | -2.411244 | 2.256011 |

| | | | |
|---|---|---|---|
| H | 1.550334 | -2.953085 | 1.122391 |
| H | -2.421874 | 0.134021 | 1.602159 |
| C | -3.386607 | -0.363773 | 1.444982 |
| H | -4.684444 | -1.836128 | -1.980159 |
| H | 2.053376 | 0.088139 | -2.584221 |
| H | 2.739960 | -0.128210 | 1.302356 |
| Br | -1.438813 | -2.594835 | -3.269589 |
| C | 1.966648 | 2.325914 | 0.088908 |
| H | 1.577975 | 3.035119 | 0.825432 |
| H | 2.770434 | 1.766317 | 0.560645 |
| H | 2.386174 | 2.883029 | -0.756065 |
| C | -4.944225 | 1.133494 | -1.729329 |
| H | -4.806661 | 1.718110 | -2.642209 |
| H | -5.542495 | 0.263969 | -1.990373 |
| H | -5.496319 | 1.736154 | -0.997936 |
| O | -0.326623 | 0.901868 | -4.070466 |
| O | -0.382921 | 0.583489 | -5.240289 |

67

Complex TSA’B

| | | | |
|---|---|---|---|
| Mo | -1.042589 | -1.113484 | -1.365503 |
| P | 1.110925 | -0.310159 | -0.483296 |
| P | -3.271808 | -0.785570 | -0.416441 |
| N | -1.359563 | 1.108788 | -0.912670 |
| N | 0.894381 | 1.417453 | -0.326363 |
| N | -3.633331 | 0.708052 | -1.262235 |
| C | -0.317018 | 1.967636 | -0.704134 |
| C | -0.470963 | 3.355342 | -0.861633 |
| C | -1.719946 | 3.855409 | -1.199435 |
| C | -2.807592 | 3.003176 | -1.349997 |
| C | -2.599779 | 1.630312 | -1.185346 |
| C | 1.875850 | -0.792630 | 1.158073 |
| C | 0.914485 | -0.492149 | 2.319391 |
| C | 2.439739 | -0.513294 | -1.819270 |
| C | 3.865448 | -0.099704 | -1.412489 |
| C | -4.485846 | 0.667490 | 1.761487 |
| C | -3.436175 | -1.581707 | 2.335876 |
| C | -4.620779 | -1.993801 | -0.912600 |
| C | -6.008082 | -1.747589 | -0.284796 |
| C | -0.901783 | -2.441569 | -0.008356 |
| O | -0.842296 | -3.294820 | 0.795705 |
| H | 0.368060 | 4.023388 | -0.736060 |
| H | -1.852773 | 4.925217 | -1.326780 |
| H | -3.793857 | 3.394471 | -1.553925 |
| H | 1.423775 | -0.687107 | 3.268524 |
| H | 0.031689 | -1.135136 | 2.278451 |
| H | 0.586432 | 0.551535 | 2.326545 |
| H | 4.521746 | -0.208854 | -2.282068 |
| H | 4.269966 | -0.739002 | -0.622894 |
| H | 3.935366 | 0.939401 | -1.086526 |
| H | -4.381722 | 0.951810 | 2.813970 |
| H | -5.488514 | 0.252233 | 1.636484 |
| H | -4.410373 | 1.583071 | 1.169325 |
| H | -3.312288 | -1.249467 | 3.371835 |
| H | -2.648584 | -2.306751 | 2.125327 |
| H | -4.400528 | -2.092332 | 2.276380 |
| H | -6.729331 | -2.405986 | -0.779605 |
| H | -6.373796 | -0.725091 | -0.387839 |
| H | -6.014872 | -2.004048 | 0.777022 |
| C | -4.182675 | -3.451795 | -0.658810 |

| | | | |
|---|---|---|---|
| H | -3.271433 | -3.711249 | -1.197146 |
| H | -4.034007 | -3.656624 | 0.404161 |
| H | -4.979357 | -4.113847 | -1.012207 |
| C | 2.436619 | -1.928384 | -2.431191 |
| H | 1.451972 | -2.236155 | -2.790764 |
| H | 3.118972 | -1.944957 | -3.287103 |
| H | 2.782932 | -2.684028 | -1.721518 |
| C | 2.370844 | -2.250938 | 1.186425 |
| H | 3.146028 | -2.444116 | 0.442125 |
| H | 2.810431 | -2.449518 | 2.169207 |
| H | 1.560935 | -2.966906 | 1.039549 |
| H | -2.414085 | 0.142677 | 1.572992 |
| C | -3.382362 | -0.347578 | 1.415554 |
| H | -4.682118 | -1.851631 | -1.999413 |
| H | 2.085911 | 0.187520 | -2.587032 |
| H | 2.757242 | -0.148544 | 1.264553 |
| Br | -1.413394 | -2.787994 | -3.157650 |
| C | 1.952603 | 2.334086 | 0.122835 |
| H | 1.558119 | 3.027464 | 0.871146 |
| H | 2.755975 | 1.768453 | 0.588051 |
| H | 2.374190 | 2.909076 | -0.709141 |
| C | -4.937307 | 1.137435 | -1.776637 |
| H | -4.786157 | 1.750249 | -2.668527 |
| H | -5.515277 | 0.267093 | -2.078721 |
| H | -5.516448 | 1.710771 | -1.042459 |
| O | -0.597106 | 0.455283 | -3.652048 |
| O | -0.158320 | 0.204622 | -4.758559 |

67

Complex B

| | | | |
|---|---|---|---|
| Mo | -1.010704 | -1.220333 | -0.444033 |
| P | 1.340339 | -0.323359 | -0.357102 |
| P | -3.468870 | -0.721378 | -0.277130 |
| N | -1.214104 | 1.083383 | -0.563100 |
| N | 1.069425 | 1.355142 | -0.001024 |
| N | -3.483527 | 0.781249 | -1.149916 |
| C | -0.155653 | 1.921714 | -0.335915 |
| C | -0.279601 | 3.316117 | -0.444241 |
| C | -1.507588 | 3.858394 | -0.786407 |
| C | -2.591888 | 3.027338 | -1.021978 |
| C | -2.416910 | 1.642838 | -0.910882 |
| C | 2.603688 | -0.875652 | 0.912607 |
| C | 2.218684 | -0.514267 | 2.356778 |
| C | 2.180737 | -0.335019 | -2.051986 |
| C | 3.596568 | 0.268730 | -2.068721 |
| C | -5.322148 | 0.620422 | 1.501751 |
| C | -4.425859 | -1.660974 | 2.221046 |
| C | -4.595921 | -1.876501 | -1.244355 |
| C | -6.111225 | -1.682665 | -1.028514 |
| C | -0.972198 | -1.491760 | 1.518814 |
| O | -1.002274 | -1.725801 | 2.655037 |
| H | 0.568884 | 3.962087 | -0.281175 |
| H | -1.620634 | 4.934971 | -0.864919 |
| H | -3.562024 | 3.441516 | -1.254705 |
| H | 3.085453 | -0.689690 | 3.002019 |
| H | 1.407038 | -1.143944 | 2.722916 |
| H | 1.920536 | 0.529677 | 2.478394 |
| H | 3.937773 | 0.330155 | -3.107300 |
| H | 4.312586 | -0.363340 | -1.534623 |
| H | 3.645958 | 1.277016 | -1.653165 |

```
 H    -5.552252     0.827729     2.552075
 H    -6.228224     0.218019     1.043496
 H    -5.083911     1.576175     1.028062
 H    -4.578060    -1.414318     3.276257
 H    -3.601939    -2.377154     2.172921
 H    -5.334029    -2.155301     1.867352
 H    -6.642210    -2.297312    -1.762605
 H    -6.456774    -0.655155    -1.150653
 H    -6.418738    -2.026486    -0.038380
 C    -4.225952    -3.356361    -1.018027
 H    -3.188208    -3.575866    -1.269112
 H    -4.404428    -3.667847     0.014910
 H    -4.862387    -3.972304    -1.660843
 C     2.174671    -1.710532    -2.744943
 H     1.189626    -2.175645    -2.774671
 H     2.510040    -1.576759    -3.778549
 H     2.866924    -2.409584    -2.268947
 C     2.916075    -2.378856     0.788010
 H     3.303101    -2.651869    -0.195642
 H     3.683823    -2.635293     1.524617
 H     2.036068    -2.993775     0.990641
 H    -3.279787     0.127022     1.910014
 C    -4.142467    -0.363120     1.440363
 H    -4.338924    -1.626058    -2.281760
 H     1.508964     0.327220    -2.615713
 H     3.515649    -0.328605     0.643447
Br    -0.422971    -3.644655    -0.619170
 C     2.163629     2.268820     0.366154
 H     1.851280     2.907208     1.197340
 H     3.026966     1.696293     0.694105
 H     2.470009     2.901131    -0.474001
 C    -4.634869     1.293353    -1.907935
 H    -4.270089     1.921744    -2.722950
 H    -5.172111     0.462380    -2.358368
 H    -5.332831     1.872478    -1.292423
 O    -1.291643    -1.337640    -2.471970
 O    -1.270159    -2.116139    -3.464966

67
Complex C
Mo     0.071737    -0.277149     0.356842
 P     2.473496     0.398075     0.084279
 P    -2.434843    -0.087607     0.226835
 N    -0.142707     1.891510    -0.002584
 N     2.233337     2.134874     0.102513
 N    -2.519960     1.622592    -0.135721
 C     0.975878     2.689870    -0.111658
 C     0.856603     4.052664    -0.415457
 C    -0.406792     4.590795    -0.600814
 C    -1.536945     3.797543    -0.505866
 C    -1.391060     2.430593    -0.218617
 C     3.787616     0.103250     1.388433
 C     3.380952     0.659884     2.760795
 C     3.229795     0.011619    -1.596661
 C     4.677794     0.486387    -1.806776
 C    -4.994067    -0.321243     1.676124
 C    -3.007711    -1.551743     2.595707
 C    -3.165077    -1.035710    -1.223106
 C    -4.486058    -0.558653    -1.850370
 C    -0.002806     0.427406     2.293849
```

| | | | |
|---|---|---|---|
| O | -0.110066 | 0.845201 | 3.359359 |
| H | 1.728623 | 4.677211 | -0.525642 |
| H | -0.511299 | 5.645378 | -0.835712 |
| H | -2.509517 | 4.234413 | -0.662705 |
| H | 4.239109 | 0.603646 | 3.437966 |
| H | 2.577844 | 0.066602 | 3.204565 |
| H | 3.057713 | 1.703628 | 2.719902 |
| H | 4.973866 | 0.271321 | -2.838730 |
| H | 5.380098 | -0.043568 | -1.155900 |
| H | 4.806812 | 1.559665 | -1.652643 |
| H | -5.420717 | -0.413795 | 2.680256 |
| H | -5.344834 | -1.180492 | 1.098984 |
| H | -5.412205 | 0.585618 | 1.237967 |
| H | -3.497211 | -1.537704 | 3.574547 |
| H | -1.931088 | -1.588059 | 2.759205 |
| H | -3.300379 | -2.478676 | 2.095663 |
| H | -4.726988 | -1.227840 | -2.683254 |
| H | -4.409037 | 0.448286 | -2.265029 |
| H | -5.328951 | -0.593958 | -1.155388 |
| C | -3.222660 | -2.534464 | -0.859750 |
| H | -2.292164 | -2.900364 | -0.416380 |
| H | -4.043947 | -2.757286 | -0.172365 |
| H | -3.398501 | -3.110252 | -1.773568 |
| C | 3.084002 | -1.480071 | -1.963917 |
| H | 2.060109 | -1.840985 | -1.853587 |
| H | 3.370946 | -1.610361 | -3.012255 |
| H | 3.740039 | -2.117755 | -1.366280 |
| C | 4.153537 | -1.390236 | 1.483318 |
| H | 4.593575 | -1.773871 | 0.560788 |
| H | 4.896232 | -1.518914 | 2.276978 |
| H | 3.285534 | -2.005446 | 1.733797 |
| H | -3.162757 | 0.582230 | 2.374599 |
| C | -3.458771 | -0.308036 | 1.802889 |
| H | -2.384099 | -0.902712 | -1.978986 |
| H | 2.571771 | 0.581002 | -2.266077 |
| H | 4.678861 | 0.639349 | 1.041439 |
| Br | 0.398116 | -2.568414 | 1.267203 |
| C | 3.377786 | 3.061739 | 0.128213 |
| H | 3.203147 | 3.857228 | 0.858010 |
| H | 4.272499 | 2.525901 | 0.433620 |
| H | 3.564808 | 3.510290 | -0.853495 |
| C | -3.822030 | 2.283801 | -0.338345 |
| H | -3.875873 | 2.749684 | -1.326851 |
| H | -4.613337 | 1.545892 | -0.279865 |
| H | -4.001165 | 3.043042 | 0.430106 |
| O | 0.038508 | -0.377850 | -1.595422 |
| O | -0.420437 | 0.137176 | -2.676223 |

67
Complex TSCD

| | | | |
|---|---|---|---|
| Mo | 0.082474 | -0.360122 | -0.023751 |
| P | 2.513982 | 0.374617 | -0.048162 |
| P | -2.437079 | -0.115182 | 0.151207 |
| N | -0.137738 | 1.832410 | -0.142561 |
| N | 2.235155 | 2.095074 | 0.043204 |
| N | -2.520062 | 1.589323 | -0.208799 |
| C | 0.973283 | 2.645983 | -0.191612 |
| C | 0.856268 | 4.018242 | -0.444412 |
| C | -0.406294 | 4.561621 | -0.625408 |
| C | -1.534906 | 3.765074 | -0.549184 |

| | | | |
|---|---|---|---|
| C | -1.386544 | 2.389742 | -0.307102 |
| C | 3.669144 | 0.008034 | 1.377964 |
| C | 3.020385 | 0.404886 | 2.712783 |
| C | 3.417573 | 0.068274 | -1.672891 |
| C | 4.890647 | 0.508501 | -1.697211 |
| C | -4.875157 | -0.384697 | 1.769832 |
| C | -2.770610 | -1.506262 | 2.598254 |
| C | -3.257642 | -1.081080 | -1.237744 |
| C | -4.605963 | -0.593079 | -1.792170 |
| C | -0.369470 | 1.340738 | 3.329460 |
| O | -0.149624 | 2.105832 | 4.138178 |
| H | 1.728105 | 4.648342 | -0.519578 |
| H | -0.510527 | 5.622447 | -0.830147 |
| H | -2.509017 | 4.205985 | -0.685118 |
| H | 3.740271 | 0.260220 | 3.524456 |
| H | 2.152493 | -0.227198 | 2.927861 |
| H | 2.704756 | 1.451767 | 2.729702 |
| H | 5.289742 | 0.348596 | -2.703973 |
| H | 5.505227 | -0.081066 | -1.010452 |
| H | 5.027012 | 1.565940 | -1.461116 |
| H | -5.239614 | -0.434444 | 2.801244 |
| H | -5.230956 | -1.283607 | 1.260154 |
| H | -5.346563 | 0.484037 | 1.307326 |
| H | -3.229797 | -1.515458 | 3.592119 |
| H | -1.688402 | -1.450444 | 2.725383 |
| H | -3.000897 | -2.462345 | 2.120997 |
| H | -4.906681 | -1.272680 | -2.596584 |
| H | -4.537157 | 0.404953 | -2.229098 |
| H | -5.404953 | -0.602654 | -1.046307 |
| C | -3.308324 | -2.572870 | -0.847591 |
| H | -2.352004 | -2.945068 | -0.470198 |
| H | -4.079639 | -2.775981 | -0.098944 |
| H | -3.558492 | -3.156812 | -1.738683 |
| C | 3.257024 | -1.392336 | -2.139671 |
| H | 2.208961 | -1.687627 | -2.211640 |
| H | 3.697598 | -1.489656 | -3.137037 |
| H | 3.773859 | -2.097275 | -1.483742 |
| C | 4.102682 | -1.469946 | 1.383892 |
| H | 4.678386 | -1.741756 | 0.496687 |
| H | 4.744032 | -1.642612 | 2.253930 |
| H | 3.244628 | -2.142570 | 1.460615 |
| H | -3.039905 | 0.608054 | 2.318146 |
| C | -3.336959 | -0.314429 | 1.802141 |
| H | -2.517652 | -0.959462 | -2.036840 |
| H | 2.844913 | 0.702081 | -2.363082 |
| H | 4.564929 | 0.620851 | 1.222878 |
| Br | 0.411403 | -2.659718 | 0.911515 |
| C | 3.363655 | 3.034033 | 0.166868 |
| H | 3.141282 | 3.795688 | 0.919279 |
| H | 4.249818 | 2.495633 | 0.493846 |
| H | 3.595340 | 3.526254 | -0.784057 |
| C | -3.826030 | 2.259338 | -0.341231 |
| H | -3.926290 | 2.732742 | -1.322517 |
| H | -4.616961 | 1.523599 | -0.247975 |
| H | -3.963417 | 3.013554 | 0.440763 |
| O | 0.055475 | -0.477247 | -1.938448 |
| O | -0.704495 | 0.245369 | -2.726899 |

67
Complex D

| | | | |
|---|---|---|---|
| Mo | 0.086598 | -0.389670 | -0.130411 |
| P | 2.511043 | 0.350262 | -0.077849 |
| P | -2.422724 | -0.143411 | 0.132101 |
| N | -0.137254 | 1.806589 | -0.143894 |
| N | 2.233519 | 2.066986 | 0.045593 |
| N | -2.517579 | 1.564045 | -0.204946 |
| C | 0.972488 | 2.623140 | -0.174104 |
| C | 0.853986 | 4.001008 | -0.391434 |
| C | -0.409515 | 4.547447 | -0.557735 |
| C | -1.537316 | 3.748386 | -0.498538 |
| C | -1.387440 | 2.367604 | -0.290305 |
| C | 3.591696 | -0.053894 | 1.395201 |
| C | 2.856502 | 0.289616 | 2.701344 |
| C | 3.477740 | 0.078984 | -1.671276 |
| C | 4.960829 | 0.483283 | -1.621920 |
| C | -4.801633 | -0.452656 | 1.820671 |
| C | -2.654289 | -1.539302 | 2.588950 |
| C | -3.282204 | -1.093617 | -1.244596 |
| C | -4.647052 | -0.604565 | -1.755931 |
| C | -0.753072 | 2.247932 | 3.338583 |
| O | 0.096998 | 2.926625 | 3.665910 |
| H | 1.725641 | 4.633167 | -0.450934 |
| H | -0.514941 | 5.612897 | -0.736253 |
| H | -2.512527 | 4.190608 | -0.622373 |
| H | 3.531732 | 0.137391 | 3.549205 |
| H | 1.993024 | -0.367950 | 2.850535 |
| H | 2.514859 | 1.327962 | 2.729676 |
| H | 5.399220 | 0.335023 | -2.614053 |
| H | 5.532060 | -0.134214 | -0.922547 |
| H | 5.111570 | 1.532230 | -1.358011 |
| H | -5.137670 | -0.505178 | 2.861523 |
| H | -5.157044 | -1.357708 | 1.321724 |
| H | -5.298281 | 0.407834 | 1.368892 |
| H | -3.088124 | -1.560354 | 3.593895 |
| H | -1.570462 | -1.458666 | 2.692497 |
| H | -2.871997 | -2.500068 | 2.114951 |
| H | -4.969600 | -1.279727 | -2.555611 |
| H | -4.592810 | 0.396221 | -2.188558 |
| H | -5.424013 | -0.620772 | -0.987211 |
| C | -3.318588 | -2.589457 | -0.868321 |
| H | -2.351423 | -2.962637 | -0.521026 |
| H | -4.068423 | -2.801180 | -0.100513 |
| H | -3.592455 | -3.165182 | -1.757783 |
| C | 3.299515 | -1.363156 | -2.186895 |
| H | 2.247761 | -1.621811 | -2.323719 |
| H | 3.791764 | -1.449592 | -3.160719 |
| H | 3.755480 | -2.101412 | -1.522193 |
| C | 4.050394 | -1.523899 | 1.378118 |
| H | 4.672073 | -1.759755 | 0.511669 |
| H | 4.652553 | -1.712847 | 2.272451 |
| H | 3.201710 | -2.212347 | 1.393867 |
| H | -2.964807 | 0.568209 | 2.318479 |
| C | -3.264749 | -0.357459 | 1.811024 |
| H | -2.565715 | -0.961367 | -2.063457 |
| H | 2.949995 | 0.747711 | -2.364422 |
| H | 4.483040 | 0.579163 | 1.313483 |
| Br | 0.416256 | -2.718149 | 0.723145 |
| C | 3.362676 | 3.000297 | 0.200843 |
| H | 3.136727 | 3.743020 | 0.970766 |
| H | 4.245499 | 2.451510 | 0.519893 |

| | | | |
|---|---|---|---|
| H | 3.601621 | 3.515065 | -0.736274 |
| C | -3.826576 | 2.234570 | -0.294698 |
| H | -3.947629 | 2.729277 | -1.263041 |
| H | -4.614478 | 1.495514 | -0.201020 |
| H | -3.947990 | 2.970725 | 0.506869 |
| O | 0.023188 | -0.404059 | -2.045070 |
| O | -0.770692 | 0.324913 | -2.790465 |

65

Complex D’

| | | | |
|---|---|---|---|
| Mo | 0.061777 | -0.337277 | -0.190094 |
| P | 2.470683 | 0.417269 | -0.049900 |
| P | -2.444275 | -0.066856 | 0.084630 |
| N | -0.173323 | 1.861262 | -0.259205 |
| N | 2.186094 | 2.138628 | 0.001797 |
| N | -2.536807 | 1.583408 | -0.472804 |
| C | 0.931657 | 2.683342 | -0.254841 |
| C | 0.808207 | 4.061324 | -0.483683 |
| C | -0.450915 | 4.598737 | -0.692928 |
| C | -1.575194 | 3.789202 | -0.677606 |
| C | -1.417222 | 2.412729 | -0.467866 |
| C | 3.470508 | 0.082731 | 1.496981 |
| C | 2.659930 | 0.468198 | 2.746219 |
| C | 3.512728 | 0.080120 | -1.577758 |
| C | 4.986762 | 0.509758 | -1.495267 |
| C | -4.354373 | 0.709158 | 2.144385 |
| C | -3.146096 | -1.527429 | 2.398329 |
| C | -3.531128 | -1.059570 | -1.098022 |
| C | -5.019586 | -1.220301 | -0.737948 |
| H | 1.676667 | 4.699767 | -0.515568 |
| H | -0.558101 | 5.664495 | -0.868447 |
| H | -2.552422 | 4.221982 | -0.821749 |
| H | 3.290595 | 0.363430 | 3.634674 |
| H | 1.798812 | -0.195894 | 2.876635 |
| H | 2.303163 | 1.501730 | 2.711363 |
| H | 5.470783 | 0.287911 | -2.451875 |
| H | 5.534983 | -0.038155 | -0.722884 |
| H | 5.111856 | 1.579439 | -1.314260 |
| H | -4.538199 | 0.697827 | 3.223783 |
| H | -5.231656 | 0.272846 | 1.661415 |
| H | -4.270133 | 1.755388 | 1.841514 |
| H | -3.304667 | -1.490312 | 3.480644 |
| H | -2.228439 | -2.092263 | 2.216182 |
| H | -3.984411 | -2.080745 | 1.967055 |
| H | -5.522907 | -1.721500 | -1.570889 |
| H | -5.543839 | -0.278390 | -0.563420 |
| H | -5.154381 | -1.849015 | 0.145332 |
| C | -2.894290 | -2.441506 | -1.354154 |
| H | -1.887983 | -2.359414 | -1.768895 |
| H | -2.845418 | -3.047202 | -0.444682 |
| H | -3.510180 | -2.982200 | -2.079801 |
| C | 3.375118 | -1.394730 | -2.007402 |
| H | 2.332168 | -1.689251 | -2.142497 |
| H | 3.881823 | -1.528263 | -2.968181 |
| H | 3.836473 | -2.082141 | -1.293639 |
| C | 3.953536 | -1.377674 | 1.567708 |
| H | 4.637944 | -1.632263 | 0.755545 |
| H | 4.496040 | -1.521488 | 2.507549 |
| H | 3.118249 | -2.082250 | 1.553076 |
| H | -2.244940 | 0.420309 | 2.376169 |

| | | | |
|---|---|---|---|
| C | -3.071104 | -0.086622 | 1.856651 |
| H | -3.445033 | -0.476370 | -2.023704 |
| H | 2.995036 | 0.690121 | -2.328055 |
| H | 4.355876 | 0.726901 | 1.437966 |
| Br | 0.333091 | -2.575603 | 0.892311 |
| C | 3.310350 | 3.077194 | 0.160187 |
| H | 3.073649 | 3.829950 | 0.917316 |
| H | 4.190384 | 2.535354 | 0.497714 |
| H | 3.559062 | 3.578717 | -0.781369 |
| C | -3.815480 | 2.177802 | -0.899806 |
| H | -3.707513 | 2.632797 | -1.888608 |
| H | -4.569420 | 1.399636 | -0.969528 |
| H | -4.174370 | 2.933041 | -0.193053 |
| O | -0.111550 | -0.406989 | -2.105012 |
| O | 0.510684 | 0.413216 | -2.916886 |

65

Complex TSD’E

| | | | |
|---|---|---|---|
| Mo | 0.034684 | -0.317383 | -0.431519 |
| P | 2.464233 | 0.431044 | -0.124538 |
| P | -2.425416 | -0.069074 | 0.026565 |
| N | -0.177382 | 1.880433 | -0.350291 |
| N | 2.189399 | 2.154713 | -0.163869 |
| N | -2.552289 | 1.621913 | -0.389504 |
| C | 0.932628 | 2.696295 | -0.402335 |
| C | 0.806178 | 4.069293 | -0.660416 |
| C | -0.458756 | 4.610286 | -0.816627 |
| C | -1.587971 | 3.813188 | -0.712919 |
| C | -1.429611 | 2.439728 | -0.484117 |
| C | 3.310793 | 0.166169 | 1.525538 |
| C | 2.382832 | 0.616557 | 2.666296 |
| C | 3.661519 | 0.035371 | -1.522122 |
| C | 5.134271 | 0.410900 | -1.285560 |
| C | -4.137770 | 0.560606 | 2.298093 |
| C | -2.952201 | -1.702134 | 2.269503 |
| C | -3.621896 | -0.972379 | -1.122975 |
| C | -5.084337 | -1.128818 | -0.665578 |
| H | 1.676829 | 4.698852 | -0.754508 |
| H | -0.568018 | 5.671224 | -1.018293 |
| H | -2.566819 | 4.253012 | -0.817374 |
| H | 2.912177 | 0.538689 | 3.621209 |
| H | 1.498869 | -0.028210 | 2.731676 |
| H | 2.051537 | 1.652872 | 2.552441 |
| H | 5.709597 | 0.134101 | -2.174911 |
| H | 5.571141 | -0.129226 | -0.440734 |
| H | 5.287475 | 1.480417 | -1.128881 |
| H | -4.222892 | 0.473924 | 3.386318 |
| H | -5.061880 | 0.166489 | 1.868688 |
| H | -4.066746 | 1.625039 | 2.062430 |
| H | -3.014047 | -1.751224 | 3.361237 |
| H | -2.065591 | -2.261277 | 1.960826 |
| H | -3.836190 | -2.207886 | 1.872324 |
| H | -5.648074 | -1.596925 | -1.478859 |
| H | -5.587479 | -0.189310 | -0.428613 |
| H | -5.168151 | -1.785350 | 0.203587 |
| C | -3.037169 | -2.353091 | -1.491004 |
| H | -2.059868 | -2.274176 | -1.969680 |
| H | -2.942218 | -3.002504 | -0.615933 |
| H | -3.715942 | -2.843988 | -2.195502 |
| C | 3.520070 | -1.442079 | -1.940938 |

| | | | |
|---|---|---|---|
| H | 2.494682 | -1.686407 | -2.224512 |
| H | 4.155323 | -1.618540 | -2.814633 |
| H | 3.840755 | -2.131905 | -1.155806 |
| C | 3.768900 | -1.290652 | 1.720231 |
| H | 4.498506 | -1.605208 | 0.971101 |
| H | 4.247937 | -1.380624 | 2.700436 |
| H | 2.926451 | -1.986358 | 1.693098 |
| H | -2.021719 | 0.231838 | 2.324584 |
| C | -2.897281 | -0.223370 | 1.839422 |
| H | -3.591549 | -0.343529 | -2.021941 |
| H | 3.255804 | 0.648497 | -2.335416 |
| H | 4.202339 | 0.805156 | 1.525212 |
| Br | 0.393048 | -2.634980 | 0.432402 |
| C | 3.313450 | 3.094153 | -0.009625 |
| H | 3.059220 | 3.878407 | 0.708767 |
| H | 4.178791 | 2.562020 | 0.377831 |
| H | 3.593535 | 3.555501 | -0.962869 |
| C | -3.863984 | 2.247863 | -0.629773 |
| H | -3.909247 | 2.672541 | -1.637330 |
| H | -4.641938 | 1.496462 | -0.543828 |
| H | -4.074925 | 3.033787 | 0.102147 |
| O | -0.391015 | -0.336439 | -2.296126 |
| O | 0.726733 | 0.079749 | -2.881663 |

65
Complex E

| | | | |
|---|---|---|---|
| Mo | 0.067587 | -0.276057 | -0.710456 |
| P | 2.416323 | 0.451237 | -0.122778 |
| P | -2.362190 | -0.116616 | -0.037001 |
| N | -0.206228 | 1.874026 | -0.405252 |
| N | 2.144217 | 2.171504 | -0.139058 |
| N | -2.563548 | 1.560391 | -0.465132 |
| C | 0.882960 | 2.711128 | -0.364242 |
| C | 0.732111 | 4.096586 | -0.519279 |
| C | -0.542064 | 4.621158 | -0.665803 |
| C | -1.654402 | 3.794932 | -0.639267 |
| C | -1.466973 | 2.412502 | -0.507001 |
| C | 3.220424 | 0.152807 | 1.543089 |
| C | 2.258086 | 0.550783 | 2.674530 |
| C | 3.639941 | 0.079014 | -1.502093 |
| C | 5.092480 | 0.527366 | -1.263263 |
| C | -3.955890 | 0.584104 | 2.278732 |
| C | -2.862049 | -1.720883 | 2.258550 |
| C | -3.570478 | -1.064907 | -1.130988 |
| C | -5.011535 | -1.247978 | -0.615830 |
| H | 1.589964 | 4.750081 | -0.539129 |
| H | -0.670920 | 5.691959 | -0.787810 |
| H | -2.643959 | 4.217108 | -0.715887 |
| H | 2.775608 | 0.469098 | 3.635536 |
| H | 1.391668 | -0.117452 | 2.707791 |
| H | 1.900913 | 1.580090 | 2.575837 |
| H | 5.693032 | 0.234753 | -2.130425 |
| H | 5.538550 | 0.046595 | -0.387426 |
| H | 5.196175 | 1.608509 | -1.156585 |
| H | -4.013531 | 0.516885 | 3.370138 |
| H | -4.906226 | 0.219126 | 1.881169 |
| H | -3.850289 | 1.641027 | 2.023032 |
| H | -2.876920 | -1.745097 | 3.352853 |
| H | -2.016193 | -2.325432 | 1.925061 |
| H | -3.785026 | -2.192056 | 1.912215 |

| | | | |
|---|---|---|---|
| H | -5.611898 | -1.676046 | -1.424922 |
| H | -5.503457 | -0.324856 | -0.302519 |
| H | -5.053033 | -1.949078 | 0.220848 |
| C | -2.973225 | -2.434151 | -1.516981 |
| H | -2.017840 | -2.331291 | -2.033909 |
| H | -2.833673 | -3.079678 | -0.645320 |
| H | -3.668299 | -2.938859 | -2.195419 |
| C | 3.577375 | -1.410377 | -1.901809 |
| H | 2.566526 | -1.720911 | -2.170945 |
| H | 4.212978 | -1.559321 | -2.780286 |
| H | 3.947617 | -2.070189 | -1.113171 |
| C | 3.732847 | -1.288058 | 1.714575 |
| H | 4.507986 | -1.545639 | 0.989976 |
| H | 4.173301 | -1.385899 | 2.711953 |
| H | 2.924944 | -2.018203 | 1.631524 |
| H | -1.852117 | 0.174041 | 2.242131 |
| C | -2.760746 | -0.254873 | 1.796905 |
| H | -3.586185 | -0.443364 | -2.035071 |
| H | 3.218571 | 0.660565 | -2.331614 |
| H | 4.088372 | 0.823219 | 1.571963 |
| Br | 0.363597 | -2.480723 | 0.432834 |
| C | 3.250128 | 3.116244 | 0.094283 |
| H | 2.979987 | 3.836693 | 0.871646 |
| H | 4.127338 | 2.573869 | 0.437488 |
| H | 3.519475 | 3.655961 | -0.819751 |
| C | -3.884751 | 2.142948 | -0.751960 |
| H | -3.874942 | 2.636377 | -1.728198 |
| H | -4.630490 | 1.355119 | -0.782922 |
| H | -4.187873 | 2.864008 | 0.014092 |
| O | -0.720994 | -0.068762 | -2.502228 |
| O | 0.705257 | 0.122556 | -2.528929 |

65
Complex CPEF

| | | | |
|---|---|---|---|
| Mo | 0.060051 | -0.293353 | -0.526337 |
| P | 2.436597 | 0.403614 | -0.065164 |
| P | -2.402012 | -0.138500 | -0.024519 |
| N | -0.203523 | 1.820027 | -0.260680 |
| N | 2.144886 | 2.124725 | 0.067015 |
| N | -2.548800 | 1.514928 | -0.570470 |
| C | 0.886112 | 2.664247 | -0.146047 |
| C | 0.727552 | 4.053150 | -0.243309 |
| C | -0.537663 | 4.579596 | -0.444467 |
| C | -1.642784 | 3.749616 | -0.546416 |
| C | -1.461656 | 2.364687 | -0.456538 |
| C | 3.455613 | 0.020808 | 1.467796 |
| C | 2.751718 | 0.496441 | 2.749817 |
| C | 3.504206 | 0.165115 | -1.596507 |
| C | 4.874967 | 0.858537 | -1.585317 |
| C | -4.148760 | 0.769036 | 2.087985 |
| C | -3.169477 | -1.574432 | 2.307166 |
| C | -3.518537 | -1.152968 | -1.145862 |
| C | -5.012073 | -1.237336 | -0.779165 |
| H | 1.579357 | 4.713577 | -0.179216 |
| H | -0.666440 | 5.653989 | -0.512371 |
| H | -2.628952 | 4.170505 | -0.673229 |
| H | 3.421316 | 0.334379 | 3.600527 |
| H | 1.838872 | -0.076502 | 2.934970 |
| H | 2.498313 | 1.559676 | 2.726214 |
| H | 5.390202 | 0.626975 | -2.523421 |

| | | | |
|---|---|---|---|
| H | 5.513240 | 0.502407 | -0.770219 |
| H | 4.797876 | 1.945164 | -1.522366 |
| H | -4.318853 | 0.756388 | 3.169308 |
| H | -5.069650 | 0.427721 | 1.608827 |
| H | -3.964268 | 1.806406 | 1.798797 |
| H | -3.278369 | -1.527435 | 3.395062 |
| H | -2.324741 | -2.229816 | 2.089833 |
| H | -4.079565 | -2.026779 | 1.906725 |
| H | -5.542115 | -1.717801 | -1.607655 |
| H | -5.490455 | -0.271899 | -0.605596 |
| H | -5.172552 | -1.853288 | 0.107929 |
| C | -2.932014 | -2.569706 | -1.318830 |
| H | -1.919596 | -2.540928 | -1.725247 |
| H | -2.923405 | -3.127580 | -0.378154 |
| H | -3.558799 | -3.123451 | -2.025035 |
| C | 3.640996 | -1.328888 | -1.958272 |
| H | 2.684404 | -1.855555 | -1.958489 |
| H | 4.056964 | -1.403150 | -2.967381 |
| H | 4.326104 | -1.851461 | -1.287391 |
| C | 3.835027 | -1.469194 | 1.563610 |
| H | 4.422485 | -1.811887 | 0.711466 |
| H | 4.442275 | -1.620513 | 2.461430 |
| H | 2.948218 | -2.098927 | 1.647425 |
| H | -2.072317 | 0.268028 | 2.276380 |
| C | -2.956212 | -0.146125 | 1.771870 |
| H | -3.404442 | -0.631876 | -2.104226 |
| H | 2.873519 | 0.628892 | -2.364049 |
| H | 4.384124 | 0.593108 | 1.345769 |
| Br | 0.261134 | -2.066789 | 1.231583 |
| C | 3.242902 | 3.069358 | 0.340145 |
| H | 2.985687 | 3.719109 | 1.182158 |
| H | 4.142189 | 2.518549 | 0.602812 |
| H | 3.468093 | 3.688232 | -0.535106 |
| C | -3.817281 | 2.090336 | -1.044071 |
| H | -3.643917 | 2.658236 | -1.961844 |
| H | -4.512724 | 1.290265 | -1.276961 |
| H | -4.282638 | 2.741219 | -0.296400 |
| O | -0.733019 | -0.220228 | -2.356363 |
| O | 0.661008 | -0.643060 | -2.311844 |

65
Complex F

| | | | |
|---|---|---|---|
| Mo | 0.068124 | -0.355524 | -0.350751 |
| P | 2.441450 | 0.347204 | 0.010155 |
| P | -2.408419 | -0.161230 | -0.011171 |
| N | -0.186715 | 1.764522 | -0.180291 |
| N | 2.143225 | 2.063490 | 0.259889 |
| N | -2.497584 | 1.464191 | -0.669299 |
| C | 0.892422 | 2.609203 | 0.031120 |
| C | 0.723710 | 4.001735 | 0.014819 |
| C | -0.532984 | 4.531046 | -0.234433 |
| C | -1.621425 | 3.703600 | -0.462852 |
| C | -1.435510 | 2.317043 | -0.431247 |
| C | 3.658489 | -0.101495 | 1.371058 |
| C | 3.240137 | 0.435327 | 2.752048 |
| C | 3.330971 | 0.238104 | -1.655044 |
| C | 4.545467 | 1.164538 | -1.823420 |
| C | -4.292949 | 0.932216 | 1.888146 |
| C | -3.435068 | -1.430778 | 2.316753 |
| C | -3.457491 | -1.237493 | -1.137080 |

| | | | |
|---|---|---|---|
| C | -4.983803 | -1.234446 | -0.924168 |
| H | 1.559551 | 4.662761 | 0.179576 |
| H | -0.666172 | 5.608425 | -0.243761 |
| H | -2.602651 | 4.122743 | -0.628072 |
| H | 4.029450 | 0.197789 | 3.472487 |
| H | 2.320071 | -0.041900 | 3.093986 |
| H | 3.092830 | 1.516838 | 2.769987 |
| H | 4.976775 | 0.993339 | -2.815355 |
| H | 5.334307 | 0.954866 | -1.093588 |
| H | 4.284551 | 2.222547 | -1.765321 |
| H | -4.570979 | 0.978013 | 2.946362 |
| H | -5.178275 | 0.609968 | 1.333740 |
| H | -4.033618 | 1.947193 | 1.576642 |
| H | -3.633312 | -1.319673 | 3.387631 |
| H | -2.603788 | -2.129647 | 2.209625 |
| H | -4.327671 | -1.869450 | 1.865146 |
| H | -5.447902 | -1.770149 | -1.758704 |
| H | -5.430831 | -0.239136 | -0.886793 |
| H | -5.265266 | -1.760141 | -0.009184 |
| C | -2.915058 | -2.682435 | -1.105669 |
| H | -1.862648 | -2.728849 | -1.390916 |
| H | -3.034379 | -3.144079 | -0.121342 |
| H | -3.482096 | -3.284909 | -1.822264 |
| C | 3.699598 | -1.213634 | -2.019065 |
| H | 2.902962 | -1.928051 | -1.801470 |
| H | 3.895217 | -1.262773 | -3.094540 |
| H | 4.611927 | -1.532892 | -1.508342 |
| C | 3.877320 | -1.627673 | 1.431816 |
| H | 4.241185 | -2.047187 | 0.493657 |
| H | 4.629566 | -1.838797 | 2.198481 |
| H | 2.958010 | -2.145971 | 1.711238 |
| H | -2.261597 | 0.360002 | 2.289236 |
| C | -3.115325 | -0.042582 | 1.729467 |
| H | -3.223421 | -0.824620 | -2.125400 |
| H | 2.543140 | 0.563235 | -2.344375 |
| H | 4.608827 | 0.364631 | 1.080652 |
| Br | 0.168641 | -1.509307 | 1.886163 |
| C | 3.238413 | 2.997650 | 0.575508 |
| H | 3.002652 | 3.573077 | 1.475678 |
| H | 4.151277 | 2.437913 | 0.760465 |
| H | 3.430054 | 3.689612 | -0.250402 |
| C | -3.695278 | 2.023313 | -1.316024 |
| H | -3.392192 | 2.663208 | -2.147955 |
| H | -4.291503 | 1.212992 | -1.726097 |
| H | -4.320526 | 2.600321 | -0.625804 |
| O | -0.648382 | -0.441189 | -2.184410 |
| O | 0.572894 | -1.229031 | -1.948830 |

68
Complex F’

| | | | |
|---|---|---|---|
| Mo | -0.341294 | -0.421922 | -0.242036 |
| P | 2.116470 | -0.042926 | -0.000409 |
| P | -2.759876 | 0.143336 | 0.119882 |
| N | -0.289752 | 1.719831 | -0.194022 |
| N | 2.066641 | 1.707867 | 0.193000 |
| N | -2.629634 | 1.723070 | -0.635360 |
| C | 0.901025 | 2.412939 | -0.037223 |
| C | 0.926197 | 3.814666 | -0.110855 |
| C | -0.249239 | 4.502713 | -0.364947 |
| C | -1.446925 | 3.826009 | -0.542007 |

| | | | |
|---|---|---|---|
| C | -1.454264 | 2.430195 | -0.451340 |
| C | 3.311309 | -0.626548 | 1.326416 |
| C | 3.060990 | 0.038080 | 2.692648 |
| C | 2.889037 | -0.326854 | -1.701390 |
| C | 4.145485 | 0.504548 | -2.005418 |
| C | -4.441092 | 1.599548 | 1.972193 |
| C | -3.913606 | -0.833478 | 2.527428 |
| C | -3.974821 | -0.832641 | -0.927639 |
| C | -5.479939 | -0.593362 | -0.699138 |
| H | 1.849098 | 4.358291 | 0.013749 |
| H | -0.232252 | 5.586905 | -0.418665 |
| H | -2.364253 | 4.370224 | -0.710236 |
| H | 3.827943 | -0.308103 | 3.393288 |
| H | 2.087271 | -0.245425 | 3.096228 |
| H | 3.114083 | 1.127769 | 2.662090 |
| H | 4.492893 | 0.241449 | -3.010595 |
| H | 4.961024 | 0.284833 | -1.310850 |
| H | 3.948835 | 1.578338 | -2.007943 |
| H | -4.700108 | 1.731484 | 3.028148 |
| H | -5.368794 | 1.385187 | 1.435216 |
| H | -4.041621 | 2.552337 | 1.615839 |
| H | -4.075774 | -0.633893 | 3.591538 |
| H | -3.188366 | -1.645985 | 2.454922 |
| H | -4.865884 | -1.170098 | 2.111710 |
| H | -6.034234 | -1.109507 | -1.489948 |
| H | -5.778290 | 0.456413 | -0.725263 |
| H | -5.815417 | -1.010982 | 0.252434 |
| C | -3.651029 | -2.337747 | -0.812429 |
| H | -2.619063 | -2.557236 | -1.091567 |
| H | -3.832035 | -2.718046 | 0.196972 |
| H | -4.305983 | -2.889911 | -1.493795 |
| C | 3.147957 | -1.822530 | -1.967374 |
| H | 2.313411 | -2.461268 | -1.667819 |
| H | 3.289013 | -1.961277 | -3.044102 |
| H | 4.060920 | -2.152458 | -1.465159 |
| C | 3.254392 | -2.163188 | 1.460870 |
| H | 3.527880 | -2.679008 | 0.541160 |
| H | 3.970602 | -2.465382 | 2.232282 |
| H | 2.264984 | -2.499772 | 1.776642 |
| H | -2.504625 | 0.769526 | 2.384103 |
| C | -3.415278 | 0.460820 | 1.855726 |
| H | -3.701805 | -0.517420 | -1.941550 |
| H | 2.082715 | -0.000731 | -2.368106 |
| H | 4.312885 | -0.367506 | 0.963062 |
| Br | -0.345193 | -1.378320 | 2.088166 |
| C | 3.293750 | 2.485604 | 0.438848 |
| H | 3.175319 | 3.116266 | 1.325118 |
| H | 4.123021 | 1.805095 | 0.613177 |
| H | 3.551060 | 3.116404 | -0.417209 |
| C | -3.749287 | 2.413279 | -1.294664 |
| H | -3.373604 | 2.971446 | -2.155357 |
| H | -4.455210 | 1.675414 | -1.665987 |
| H | -4.281083 | 3.097795 | -0.624578 |
| O | -1.111487 | -0.530266 | -2.051172 |
| O | -0.015987 | -1.475860 | -1.778121 |
| O | 6.226252 | -1.543694 | -0.141801 |
| H | 6.859763 | -1.579455 | -0.869594 |
| H | 6.593857 | -2.136634 | 0.525706 |

68

Complex TSF’G

| | | | |
|---|---|---|---|
| Mo | -0.165859 | -0.267098 | -0.223434 |
| P | 2.254936 | 0.313492 | 0.046661 |
| P | -2.628793 | 0.079040 | 0.111257 |
| N | -0.297025 | 1.867130 | -0.178025 |
| N | 2.051580 | 2.057189 | 0.216957 |
| N | -2.628635 | 1.672289 | -0.626960 |
| C | 0.830377 | 2.658972 | -0.021434 |
| C | 0.737051 | 4.057417 | -0.102766 |
| C | -0.491978 | 4.642490 | -0.361246 |
| C | -1.627503 | 3.866276 | -0.536992 |
| C | -1.517068 | 2.475062 | -0.440840 |
| C | 3.474915 | -0.145991 | 1.399294 |
| C | 3.166071 | 0.522379 | 2.751382 |
| C | 3.100850 | 0.081886 | -1.628480 |
| C | 4.361874 | 0.928714 | -1.859562 |
| C | -4.467748 | 1.346248 | 1.953149 |
| C | -3.706522 | -1.027114 | 2.494131 |
| C | -3.740777 | -0.988290 | -0.963594 |
| C | -5.263333 | -0.895479 | -0.744323 |
| H | 1.609875 | 4.678412 | 0.020449 |
| H | -0.566623 | 5.723966 | -0.420110 |
| H | -2.586654 | 4.330854 | -0.710301 |
| H | 3.946196 | 0.237659 | 3.464844 |
| H | 2.209616 | 0.176954 | 3.148112 |
| H | 3.144926 | 1.612685 | 2.706418 |
| H | 4.761657 | 0.687470 | -2.850070 |
| H | 5.152434 | 0.705043 | -1.136005 |
| H | 4.163661 | 2.001620 | -1.846085 |
| H | -4.745169 | 1.448630 | 3.007626 |
| H | -5.365405 | 1.039915 | 1.409801 |
| H | -4.164299 | 2.335172 | 1.600874 |
| H | -3.893140 | -0.857793 | 3.559462 |
| H | -2.904982 | -1.763793 | 2.416988 |
| H | -4.619121 | -1.450423 | 2.068158 |
| H | -5.760998 | -1.447130 | -1.548676 |
| H | -5.657408 | 0.122558 | -0.755420 |
| H | -5.564754 | -1.359401 | 0.197231 |
| C | -3.276238 | -2.457924 | -0.871994 |
| H | -2.230686 | -2.574253 | -1.162590 |
| H | -3.411118 | -2.868110 | 0.132940 |
| H | -3.882241 | -3.060023 | -1.556443 |
| C | 3.381066 | -1.402958 | -1.925329 |
| H | 2.537020 | -2.053119 | -1.689451 |
| H | 3.583664 | -1.505000 | -2.996549 |
| H | 4.255994 | -1.773239 | -1.387335 |
| C | 3.547468 | -1.678683 | 1.565892 |
| H | 3.774086 | -2.221193 | 0.647774 |
| H | 4.341502 | -1.903906 | 2.285944 |
| H | 2.610092 | -2.065941 | 1.970323 |
| H | -2.461743 | 0.709417 | 2.380692 |
| C | -3.332160 | 0.316949 | 1.840302 |
| H | -3.492562 | -0.631332 | -1.969883 |
| H | 2.319085 | 0.421399 | -2.318180 |
| H | 4.451539 | 0.204658 | 1.040700 |
| Br | -0.105944 | -1.192317 | 2.117286 |
| C | 3.204441 | 2.943218 | 0.455681 |
| H | 3.025106 | 3.577707 | 1.328796 |
| H | 4.089254 | 2.342691 | 0.649051 |
| H | 3.412529 | 3.578483 | -0.411003 |

| | | | |
|---|---|---|---|
| C | -3.799099 | 2.267686 | -1.290510 |
| H | -3.469345 | 2.844702 | -2.157624 |
| H | -4.447499 | 1.474212 | -1.651442 |
| H | -4.380807 | 2.915880 | -0.625808 |
| O | -0.911950 | -0.414969 | -2.042204 |
| O | 0.261096 | -1.262229 | -1.773783 |
| O | 4.168236 | -4.286225 | -0.539765 |
| H | 4.014973 | -4.789043 | -1.349106 |
| H | 4.468995 | -4.945298 | 0.098039 |

68
Complex G

| | | | |
|---|---|---|---|
| Mo | 0.044777 | -0.317522 | -0.190029 |
| P | 2.445468 | 0.362761 | 0.021851 |
| P | -2.449151 | -0.089143 | 0.093839 |
| N | -0.161822 | 1.837139 | -0.160714 |
| N | 2.200314 | 2.093089 | 0.101266 |
| N | -2.515438 | 1.569974 | -0.463830 |
| C | 0.941183 | 2.656810 | -0.057784 |
| C | 0.806497 | 4.052854 | -0.118023 |
| C | -0.453645 | 4.601928 | -0.285794 |
| C | -1.571453 | 3.790229 | -0.395111 |
| C | -1.408667 | 2.400466 | -0.329481 |
| C | 3.723665 | -0.044509 | 1.348082 |
| C | 3.636526 | 0.795462 | 2.634946 |
| C | 3.283545 | 0.033202 | -1.639755 |
| C | 4.500689 | 0.905853 | -1.983252 |
| C | -4.610145 | 0.644368 | 1.911022 |
| C | -3.400136 | -1.574613 | 2.280191 |
| C | -3.438845 | -1.084513 | -1.176282 |
| C | -4.966546 | -1.186621 | -1.002052 |
| H | 1.666018 | 4.699053 | -0.040967 |
| H | -0.566974 | 5.680803 | -0.325863 |
| H | -2.551021 | 4.229090 | -0.504700 |
| H | 4.433742 | 0.469292 | 3.311278 |
| H | 2.682636 | 0.644629 | 3.141856 |
| H | 3.770363 | 1.864631 | 2.468906 |
| H | 4.896546 | 0.580215 | -2.951033 |
| H | 5.314542 | 0.802459 | -1.257564 |
| H | 4.247830 | 1.962899 | -2.079499 |
| H | -4.922612 | 0.606925 | 2.960225 |
| H | -5.419014 | 0.215866 | 1.316201 |
| H | -4.502025 | 1.698676 | 1.647414 |
| H | -3.721358 | -1.560587 | 3.326397 |
| H | -2.439504 | -2.096159 | 2.244708 |
| H | -4.136274 | -2.158242 | 1.720915 |
| H | -5.377374 | -1.702100 | -1.876482 |
| H | -5.477362 | -0.225058 | -0.927512 |
| H | -5.239284 | -1.776762 | -0.123681 |
| C | -2.842232 | -2.502456 | -1.303150 |
| H | -1.801089 | -2.470698 | -1.628794 |
| H | -2.922216 | -3.063464 | -0.365478 |
| H | -3.404574 | -3.058828 | -2.059809 |
| C | 3.627587 | -1.461326 | -1.787151 |
| H | 2.797364 | -2.115468 | -1.511316 |
| H | 3.870106 | -1.663734 | -2.834944 |
| H | 4.504850 | -1.732920 | -1.192297 |
| C | 3.649216 | -1.545230 | 1.702627 |
| H | 3.668045 | -2.201306 | 0.831446 |
| H | 4.509261 | -1.800539 | 2.329879 |

| | | | |
|---|---|---|---|
| H | 2.742975 | -1.756386 | 2.274391 |
| H | -2.541776 | 0.377470 | 2.395381 |
| C | -3.287373 | -0.125177 | 1.770490 |
| H | -3.213978 | -0.553579 | -2.107963 |
| H | 2.478781 | 0.260058 | -2.346682 |
| H | 4.692555 | 0.144086 | 0.867605 |
| Br | 0.100775 | -0.220130 | 2.425737 |
| C | 3.342842 | 3.016047 | 0.210224 |
| H | 3.230518 | 3.670180 | 1.080015 |
| H | 4.258580 | 2.443952 | 0.333103 |
| H | 3.447128 | 3.631443 | -0.689028 |
| C | -3.747507 | 2.179425 | -0.989873 |
| H | -3.525775 | 2.727469 | -1.909837 |
| H | -4.461540 | 1.397909 | -1.229877 |
| H | -4.218621 | 2.859536 | -0.272389 |
| O | -0.625927 | -0.233173 | -2.031766 |
| O | 0.487600 | -1.167331 | -1.860168 |
| O | 0.457727 | -2.545976 | 0.405498 |
| H | -0.044198 | -3.188859 | -0.116154 |
| H | 0.260184 | -2.695590 | 1.343483 |

68

Complex TSGH

| | | | |
|---|---|---|---|
| Mo | 0.066137 | -0.407875 | -0.153452 |
| P | 2.459983 | 0.351093 | 0.048393 |
| P | -2.457759 | -0.151723 | 0.121522 |
| N | -0.167042 | 1.775079 | -0.210303 |
| N | 2.172861 | 2.072499 | 0.149652 |
| N | -2.500074 | 1.452818 | -0.588573 |
| C | 0.910859 | 2.616165 | -0.062941 |
| C | 0.751208 | 4.009842 | -0.123666 |
| C | -0.514519 | 4.534429 | -0.328249 |
| C | -1.611406 | 3.698692 | -0.473266 |
| C | -1.418067 | 2.313475 | -0.410673 |
| C | 3.699968 | -0.087161 | 1.401658 |
| C | 3.605600 | 0.726580 | 2.704779 |
| C | 3.379289 | 0.077119 | -1.579913 |
| C | 4.579244 | 1.000450 | -1.845680 |
| C | -4.495692 | 0.842941 | 1.943417 |
| C | -3.374898 | -1.398849 | 2.469932 |
| C | -3.539871 | -1.210980 | -1.010788 |
| C | -5.063800 | -1.189503 | -0.777613 |
| H | 1.594146 | 4.673243 | -0.013872 |
| H | -0.649047 | 5.610960 | -0.364720 |
| H | -2.599710 | 4.113879 | -0.601023 |
| H | 4.398420 | 0.383753 | 3.377959 |
| H | 2.649287 | 0.567667 | 3.203113 |
| H | 3.743196 | 1.798875 | 2.563034 |
| H | 5.037700 | 0.704637 | -2.795210 |
| H | 5.356458 | 0.916922 | -1.078861 |
| H | 4.290908 | 2.048212 | -1.941735 |
| H | -4.754338 | 0.935632 | 3.003747 |
| H | -5.354378 | 0.397786 | 1.437675 |
| H | -4.348656 | 1.855491 | 1.559842 |
| H | -3.633657 | -1.276285 | 3.526446 |
| H | -2.445396 | -1.972935 | 2.427152 |
| H | -4.173418 | -1.984175 | 2.006069 |
| H | -5.544642 | -1.741778 | -1.591705 |
| H | -5.501971 | -0.190594 | -0.758239 |
| H | -5.334751 | -1.689990 | 0.155095 |

| | | | |
|---|---|---|---|
| C | -3.043132 | -2.671528 | -1.001050 |
| H | -2.014517 | -2.749299 | -1.354646 |
| H | -3.119119 | -3.124795 | -0.007684 |
| H | -3.668326 | -3.263872 | -1.676419 |
| C | 3.792161 | -1.399323 | -1.740374 |
| H | 2.994658 | -2.103045 | -1.488735 |
| H | 4.072897 | -1.576200 | -2.783225 |
| H | 4.663424 | -1.639792 | -1.123990 |
| C | 3.603989 | -1.596614 | 1.717919 |
| H | 3.652151 | -2.231096 | 0.832075 |
| H | 4.441884 | -1.869963 | 2.367208 |
| H | 2.676748 | -1.824618 | 2.247305 |
| H | -2.426960 | 0.512549 | 2.374258 |
| C | -3.220887 | -0.007266 | 1.828929 |
| H | -3.319544 | -0.798707 | -2.002632 |
| H | 2.607154 | 0.295373 | -2.323912 |
| H | 4.679009 | 0.101945 | 0.942159 |
| Br | 0.129848 | -0.279373 | 2.379983 |
| C | 3.288965 | 3.016536 | 0.332229 |
| H | 3.115748 | 3.650217 | 1.207163 |
| H | 4.209964 | 2.462641 | 0.491645 |
| H | 3.426124 | 3.651860 | -0.548516 |
| C | -3.700651 | 2.016639 | -1.227249 |
| H | -3.402542 | 2.614549 | -2.091831 |
| H | -4.331359 | 1.209813 | -1.588688 |
| H | -4.294204 | 2.640229 | -0.549595 |
| O | -0.671017 | -0.564456 | -1.943181 |
| O | 0.675257 | -1.033804 | -2.143748 |
| O | 0.406600 | -2.472267 | -0.366106 |
| H | 0.605378 | -2.038573 | -1.600583 |
| H | -0.286897 | -3.115902 | -0.174822 |

68

Complex H

| | | | |
|---|---|---|---|
| Mo | 0.073534 | -0.446222 | -0.194587 |
| P | 2.451738 | 0.363496 | 0.043885 |
| P | -2.445656 | -0.153699 | 0.124590 |
| N | -0.164159 | 1.786794 | -0.225879 |
| N | 2.169828 | 2.079679 | 0.135098 |
| N | -2.494331 | 1.446432 | -0.587481 |
| C | 0.903797 | 2.627335 | -0.068103 |
| C | 0.742180 | 4.021758 | -0.112341 |
| C | -0.527206 | 4.542617 | -0.306407 |
| C | -1.620126 | 3.701559 | -0.454401 |
| C | -1.414515 | 2.316977 | -0.409521 |
| C | 3.679390 | -0.086894 | 1.405510 |
| C | 3.600386 | 0.739245 | 2.701671 |
| C | 3.385954 | 0.070843 | -1.573064 |
| C | 4.633721 | 0.939071 | -1.805699 |
| C | -4.488635 | 0.820379 | 1.941445 |
| C | -3.347929 | -1.409796 | 2.476971 |
| C | -3.518366 | -1.228941 | -1.001621 |
| C | -5.044175 | -1.209875 | -0.781471 |
| H | 1.582961 | 4.687340 | 0.002935 |
| H | -0.667729 | 5.618707 | -0.330373 |
| H | -2.612339 | 4.111262 | -0.571125 |
| H | 4.385013 | 0.385089 | 3.378665 |
| H | 2.639821 | 0.604965 | 3.198780 |
| H | 3.761370 | 1.806989 | 2.550565 |
| H | 5.092592 | 0.636870 | -2.753086 |

| | | | |
|---|---|---|---|
| H | 5.394077 | 0.806132 | -1.029017 |
| H | 4.397841 | 2.000974 | -1.888182 |
| H | -4.751583 | 0.911682 | 3.000835 |
| H | -5.341839 | 0.367806 | 1.432956 |
| H | -4.348467 | 1.833720 | 1.557568 |
| H | -3.617663 | -1.286604 | 3.530689 |
| H | -2.410261 | -1.971487 | 2.445794 |
| H | -4.134619 | -2.007223 | 2.008359 |
| H | -5.516007 | -1.774834 | -1.592227 |
| H | -5.486264 | -0.212556 | -0.778565 |
| H | -5.320560 | -1.699823 | 0.155273 |
| C | -3.018556 | -2.688202 | -0.967687 |
| H | -1.967959 | -2.769278 | -1.248803 |
| H | -3.155304 | -3.142168 | 0.018785 |
| H | -3.602211 | -3.279771 | -1.680061 |
| C | 3.729715 | -1.421988 | -1.749826 |
| H | 2.899068 | -2.091321 | -1.507538 |
| H | 4.015681 | -1.600941 | -2.791211 |
| H | 4.581888 | -1.710681 | -1.127624 |
| C | 3.558630 | -1.591960 | 1.733413 |
| H | 3.578372 | -2.233451 | 0.851213 |
| H | 4.401184 | -1.878012 | 2.371100 |
| H | 2.635309 | -1.795347 | 2.279444 |
| H | -2.417854 | 0.509886 | 2.378523 |
| C | -3.205501 | -0.018666 | 1.833115 |
| H | -3.290670 | -0.827328 | -1.996220 |
| H | 2.641293 | 0.344259 | -2.326762 |
| H | 4.661174 | 0.082256 | 0.944488 |
| Br | 0.126305 | -0.209196 | 2.354673 |
| C | 3.287693 | 3.020776 | 0.317628 |
| H | 3.117256 | 3.654547 | 1.193082 |
| H | 4.208650 | 2.466249 | 0.476076 |
| H | 3.423322 | 3.656740 | -0.563214 |
| C | -3.697013 | 2.002524 | -1.228088 |
| H | -3.398904 | 2.611985 | -2.084566 |
| H | -4.316473 | 1.192755 | -1.602891 |
| H | -4.302764 | 2.613907 | -0.549878 |
| O | -0.719048 | -0.462430 | -1.997407 |
| O | 0.638640 | -0.861832 | -2.302847 |
| O | 0.380187 | -2.402547 | -0.278033 |
| H | 0.592953 | -1.848593 | -2.262504 |
| H | -0.236861 | -2.989685 | 0.180070 |

68
Complex TSHI

| | | | |
|---|---|---|---|
| Mo | 0.074351 | -0.421360 | -0.107908 |
| P | 2.428125 | 0.387646 | 0.098021 |
| P | -2.427187 | -0.102358 | 0.187663 |
| N | -0.143923 | 1.843771 | -0.195302 |
| N | 2.207116 | 2.106584 | 0.035227 |
| N | -2.506524 | 1.546367 | -0.379026 |
| C | 0.944715 | 2.669040 | -0.146193 |
| C | 0.814552 | 4.060194 | -0.274983 |
| C | -0.453017 | 4.595786 | -0.434111 |
| C | -1.568538 | 3.773862 | -0.463447 |
| C | -1.390845 | 2.387932 | -0.339116 |
| C | 3.636673 | 0.015501 | 1.497922 |
| C | 3.591019 | 0.965826 | 2.707860 |
| C | 3.335111 | -0.107695 | -1.484910 |
| C | 4.608348 | 0.693497 | -1.811478 |

| | | | |
|---|---|---|---|
| C | -4.581603 | 0.551022 | 2.051855 |
| C | -3.271600 | -1.623685 | 2.377363 |
| C | -3.431642 | -1.115250 | -1.068674 |
| C | -4.957756 | -1.209811 | -0.868342 |
| H | 1.675054 | 4.709886 | -0.250852 |
| H | -0.574728 | 5.670539 | -0.525970 |
| H | -2.552308 | 4.205789 | -0.558955 |
| H | 4.364818 | 0.650625 | 3.415889 |
| H | 2.627837 | 0.915121 | 3.215237 |
| H | 3.791442 | 2.007043 | 2.452766 |
| H | 5.044281 | 0.280501 | -2.727389 |
| H | 5.373409 | 0.612484 | -1.032111 |
| H | 4.406662 | 1.749037 | -2.000066 |
| H | -4.853043 | 0.509731 | 3.112172 |
| H | -5.394636 | 0.085617 | 1.491912 |
| H | -4.525796 | 1.606832 | 1.778245 |
| H | -3.566316 | -1.628442 | 3.431362 |
| H | -2.290077 | -2.102186 | 2.313739 |
| H | -3.998798 | -2.234248 | 1.834334 |
| H | -5.384266 | -1.720250 | -1.738291 |
| H | -5.466215 | -0.249058 | -0.780535 |
| H | -5.214244 | -1.804415 | 0.011825 |
| C | -2.865807 | -2.544645 | -1.194815 |
| H | -1.830672 | -2.542730 | -1.537553 |
| H | -2.932563 | -3.096223 | -0.251412 |
| H | -3.455049 | -3.096974 | -1.933617 |
| C | 3.627537 | -1.620948 | -1.507554 |
| H | 2.764415 | -2.227378 | -1.221495 |
| H | 3.918004 | -1.905338 | -2.524190 |
| H | 4.464784 | -1.876509 | -0.851329 |
| C | 3.461944 | -1.446830 | 1.964788 |
| H | 3.473253 | -2.168483 | 1.146554 |
| H | 4.286361 | -1.697221 | 2.640138 |
| H | 2.524970 | -1.567307 | 2.512052 |
| H | -2.496142 | 0.363365 | 2.484185 |
| C | -3.234489 | -0.167529 | 1.875772 |
| H | -3.218983 | -0.587955 | -2.005438 |
| H | 2.591938 | 0.105289 | -2.257833 |
| H | 4.622342 | 0.105896 | 1.022786 |
| Br | 0.101829 | 0.049775 | 2.443464 |
| C | 3.357158 | 3.022499 | 0.106214 |
| H | 3.239929 | 3.727135 | 0.934972 |
| H | 4.266523 | 2.450844 | 0.273318 |
| H | 3.476919 | 3.583245 | -0.826435 |
| C | -3.769747 | 2.147210 | -0.837225 |
| H | -3.615270 | 2.652974 | -1.794666 |
| H | -4.508616 | 1.367347 | -0.987171 |
| H | -4.176580 | 2.863656 | -0.115741 |
| O | -0.605161 | -0.332301 | -1.926740 |
| O | 0.538389 | -0.784210 | -2.719523 |
| O | 0.409215 | -2.309344 | -0.219073 |
| H | 0.243320 | -1.667648 | -3.006972 |
| H | -0.260221 | -3.008888 | -0.216304 |

68

Complex I

| | | | |
|---|---|---|---|
| Mo | 0.052315 | -0.514446 | 0.003634 |
| P | 2.397661 | 0.325864 | 0.147489 |
| P | -2.433739 | -0.113557 | 0.191582 |
| N | -0.166890 | 1.748343 | -0.141596 |

| | | | |
|---|---|---|---|
| N | 2.158253 | 2.042637 | 0.246886 |
| N | -2.480567 | 1.437165 | -0.601531 |
| C | 0.903376 | 2.591209 | 0.020760 |
| C | 0.755612 | 3.985451 | -0.056984 |
| C | -0.496012 | 4.513450 | -0.326325 |
| C | -1.585323 | 3.677244 | -0.515526 |
| C | -1.399508 | 2.292986 | -0.415424 |
| C | 3.657044 | -0.146745 | 1.466493 |
| C | 3.598822 | 0.658142 | 2.777424 |
| C | 3.235279 | 0.009943 | -1.518947 |
| C | 4.305877 | 1.039333 | -1.918588 |
| C | -4.512453 | 0.967400 | 1.901017 |
| C | -3.434120 | -1.258984 | 2.558974 |
| C | -3.409368 | -1.298584 | -0.899443 |
| C | -4.945294 | -1.279745 | -0.769980 |
| H | 1.598586 | 4.643167 | 0.083311 |
| H | -0.625209 | 5.589872 | -0.381272 |
| H | -2.567119 | 4.089622 | -0.694453 |
| H | 4.412095 | 0.309710 | 3.422866 |
| H | 2.656676 | 0.491082 | 3.299152 |
| H | 3.731317 | 1.731673 | 2.641446 |
| H | 4.705768 | 0.758892 | -2.898615 |
| H | 5.152226 | 1.057975 | -1.223690 |
| H | 3.903589 | 2.049678 | -2.011739 |
| H | -4.828867 | 1.078624 | 2.943524 |
| H | -5.346801 | 0.521667 | 1.354582 |
| H | -4.334781 | 1.972629 | 1.510855 |
| H | -3.706042 | -1.086087 | 3.604999 |
| H | -2.516052 | -1.851582 | 2.557591 |
| H | -4.238188 | -1.845053 | 2.105948 |
| H | -5.365243 | -1.909381 | -1.561425 |
| H | -5.394355 | -0.290420 | -0.868972 |
| H | -5.272419 | -1.701023 | 0.183305 |
| C | -2.899902 | -2.744575 | -0.715354 |
| H | -1.828755 | -2.839615 | -0.897903 |
| H | -3.108803 | -3.124313 | 0.288443 |
| H | -3.420467 | -3.394327 | -1.426270 |
| C | 3.795380 | -1.419437 | -1.633665 |
| H | 3.071531 | -2.182441 | -1.342217 |
| H | 4.070940 | -1.601878 | -2.677237 |
| H | 4.699882 | -1.548312 | -1.031788 |
| C | 3.547743 | -1.656682 | 1.775133 |
| H | 3.613458 | -2.288080 | 0.889344 |
| H | 4.370258 | -1.932097 | 2.442839 |
| H | 2.606737 | -1.877360 | 2.281843 |
| H | -2.455440 | 0.631875 | 2.416364 |
| C | -3.242508 | 0.103382 | 1.866887 |
| H | -3.120080 | -0.966296 | -1.903744 |
| H | 2.397713 | 0.095195 | -2.220248 |
| H | 4.625784 | 0.037268 | 0.980949 |
| Br | 0.126694 | -0.367269 | 2.591470 |
| C | 3.286728 | 2.969585 | 0.436734 |
| H | 3.128414 | 3.589959 | 1.323869 |
| H | 4.202495 | 2.401697 | 0.576958 |
| H | 3.423439 | 3.616906 | -0.434715 |
| C | -3.659269 | 1.967070 | -1.302985 |
| H | -3.331961 | 2.549192 | -2.167801 |
| H | -4.260223 | 1.140762 | -1.673507 |
| H | -4.291385 | 2.596705 | -0.667050 |
| O | -0.466030 | -0.580220 | -1.865153 |

| | | | |
|---|---|---|---|
| O | 0.448361 | -1.256630 | -2.818976 |
| O | 0.746311 | -2.237274 | -0.360460 |
| H | -0.124231 | -1.297573 | -3.602979 |
| H | 0.630022 | -2.562123 | -1.274716 |

68

Complex J

| | | | |
|---|---|---|---|
| Mo | 0.095491 | -0.573490 | 0.367270 |
| P | 2.394391 | 0.352450 | -0.020741 |
| P | -2.395573 | -0.135697 | 0.356405 |
| N | -0.195862 | 1.707105 | -0.127060 |
| N | 2.051451 | 1.829434 | -0.853562 |
| N | -2.425670 | 1.565352 | 0.656628 |
| C | 0.826868 | 2.450456 | -0.648310 |
| C | 0.668579 | 3.812053 | -0.951772 |
| C | -0.555549 | 4.414852 | -0.709104 |
| C | -1.601944 | 3.679775 | -0.173265 |
| C | -1.394245 | 2.324756 | 0.113015 |
| C | 3.469048 | 0.826047 | 1.442000 |
| C | 2.949626 | 2.062501 | 2.189357 |
| C | 3.520150 | -0.642064 | -1.181556 |
| C | 5.032323 | -0.410318 | -0.996659 |
| C | -4.870482 | -0.979394 | 1.627317 |
| C | -2.817874 | -2.422240 | 1.945842 |
| C | -3.213495 | -0.386578 | -1.311462 |
| C | -4.508778 | 0.406218 | -1.546847 |
| H | 1.491278 | 4.392208 | -1.340599 |
| H | -0.694895 | 5.466085 | -0.941027 |
| H | -2.568710 | 4.133277 | -0.009572 |
| H | 3.661035 | 2.318504 | 2.981653 |
| H | 1.981265 | 1.870057 | 2.654978 |
| H | 2.859557 | 2.934203 | 1.534583 |
| H | 5.561066 | -0.926314 | -1.804518 |
| H | 5.395776 | -0.828118 | -0.053715 |
| H | 5.333235 | 0.639545 | -1.033794 |
| H | -5.283577 | -1.379087 | 2.559184 |
| H | -5.216066 | -1.626868 | 0.817600 |
| H | -5.304961 | 0.010544 | 1.476412 |
| H | -3.338246 | -2.856791 | 2.805019 |
| H | -1.748507 | -2.451344 | 2.158709 |
| H | -3.027322 | -3.059181 | 1.081268 |
| H | -4.810939 | 0.285757 | -2.592735 |
| H | -4.369368 | 1.475862 | -1.371081 |
| H | -5.336833 | 0.055916 | -0.926925 |
| C | -3.364659 | -1.881994 | -1.643303 |
| H | -2.427292 | -2.428374 | -1.508566 |
| H | -4.140350 | -2.362714 | -1.041184 |
| H | -3.658532 | -1.987616 | -2.692720 |
| C | 3.218104 | -2.152247 | -1.124813 |
| H | 2.202551 | -2.364034 | -1.461677 |
| H | 3.902027 | -2.672299 | -1.803158 |
| H | 3.374230 | -2.568800 | -0.124681 |
| C | 3.660490 | -0.382145 | 2.378317 |
| H | 4.063500 | -1.258638 | 1.861562 |
| H | 4.370558 | -0.114879 | 3.167360 |
| H | 2.717358 | -0.663345 | 2.852638 |
| H | -3.019341 | -0.397960 | 2.615293 |
| C | -3.334320 | -0.981863 | 1.742717 |
| H | -2.442210 | 0.017595 | -1.977942 |
| H | 3.204820 | -0.284883 | -2.166842 |

| | | | |
|---|---|---|---|
| H | 4.438737 | 1.079117 | 0.996993 |
| Br | -0.120933 | 0.159161 | 2.868885 |
| C | 3.107464 | 2.582156 | -1.542754 |
| H | 3.498903 | 3.403439 | -0.930651 |
| H | 3.928375 | 1.913644 | -1.787848 |
| H | 2.723118 | 2.986140 | -2.482916 |
| C | -3.525228 | 2.260651 | 1.341034 |
| H | -4.281614 | 2.648105 | 0.648730 |
| H | -4.007017 | 1.579005 | 2.038118 |
| H | -3.114850 | 3.086088 | 1.926428 |
| O | -0.232208 | -0.911056 | -1.486681 |
| O | 0.793241 | -0.576083 | -2.538231 |
| O | 0.588058 | -2.299684 | 0.898153 |
| H | 0.267935 | -0.759219 | -3.334654 |
| H | 0.762319 | -3.021987 | 0.275345 |

68
Complex TSJK

| | | | |
|---|---|---|---|
| Mo | 0.046662 | -0.435187 | 0.161693 |
| P | 2.431890 | 0.303711 | 0.171137 |
| P | -2.441358 | -0.159341 | 0.343904 |
| N | -0.174120 | 1.733960 | -0.149023 |
| N | 2.204207 | 1.994366 | -0.152670 |
| N | -2.558612 | 1.528717 | -0.044940 |
| C | 0.937988 | 2.522922 | -0.356599 |
| C | 0.813501 | 3.867690 | -0.736720 |
| C | -0.449422 | 4.412554 | -0.878047 |
| C | -1.577589 | 3.644232 | -0.644040 |
| C | -1.424421 | 2.298139 | -0.283615 |
| C | 3.651241 | 0.230349 | 1.602805 |
| C | 3.418455 | 1.293983 | 2.690684 |
| C | 3.308643 | -0.441836 | -1.332628 |
| C | 4.619317 | 0.251215 | -1.743803 |
| C | -4.713311 | 0.089718 | 2.224264 |
| C | -3.170620 | -1.910800 | 2.404896 |
| C | -3.242719 | -1.114846 | -1.076048 |
| C | -4.695120 | -0.762662 | -1.444642 |
| H | 1.684847 | 4.473940 | -0.925016 |
| H | -0.558286 | 5.451641 | -1.172622 |
| H | -2.555084 | 4.086052 | -0.746815 |
| H | 4.222695 | 1.214395 | 3.429508 |
| H | 2.470608 | 1.130794 | 3.204958 |
| H | 3.428546 | 2.315242 | 2.305468 |
| H | 5.034164 | -0.284188 | -2.604376 |
| H | 5.379740 | 0.221630 | -0.956468 |
| H | 4.470898 | 1.286772 | -2.052999 |
| H | -5.019441 | -0.191151 | 3.237653 |
| H | -5.437114 | -0.357235 | 1.536851 |
| H | -4.789187 | 1.176442 | 2.161821 |
| H | -3.337831 | -1.990671 | 3.483260 |
| H | -2.186105 | -2.336910 | 2.197479 |
| H | -3.929466 | -2.520693 | 1.908979 |
| H | -4.998189 | -1.404048 | -2.279117 |
| H | -4.813203 | 0.268971 | -1.778107 |
| H | -5.393158 | -0.953936 | -0.624904 |
| C | -3.097470 | -2.640514 | -0.897459 |
| H | -2.103304 | -2.943974 | -0.563282 |
| H | -3.836159 | -3.036900 | -0.197150 |
| H | -3.279289 | -3.118066 | -1.865565 |
| C | 3.527436 | -1.961087 | -1.190017 |

| | | | |
|---|---|---|---|
| H | 2.648306 | -2.491668 | -0.816566 |
| H | 3.760141 | -2.369374 | -2.178864 |
| H | 4.377812 | -2.188405 | -0.541533 |
| C | 3.678444 | -1.181936 | 2.224956 |
| H | 3.890372 | -1.969122 | 1.500185 |
| H | 4.468387 | -1.213440 | 2.982098 |
| H | 2.731110 | -1.409666 | 2.717010 |
| H | -2.609905 | 0.138867 | 2.658489 |
| C | -3.280944 | -0.425360 | 2.001785 |
| H | -2.592098 | -0.805020 | -1.903132 |
| H | 2.570049 | -0.269308 | -2.120658 |
| H | 4.630108 | 0.413523 | 1.140277 |
| Br | 0.109634 | -0.305048 | 2.694365 |
| C | 3.353707 | 2.908975 | -0.255366 |
| H | 3.229391 | 3.760814 | 0.419288 |
| H | 4.260275 | 2.382409 | 0.032197 |
| H | 3.483537 | 3.276706 | -1.278305 |
| C | -3.865130 | 2.197093 | -0.177589 |
| H | -4.009798 | 2.576654 | -1.194246 |
| H | -4.656787 | 1.486630 | 0.031569 |
| H | -3.954253 | 3.025800 | 0.531949 |
| O | -0.349676 | -0.255827 | -2.036964 |
| O | 0.634667 | 0.520521 | -2.788380 |
| O | 0.201290 | -2.034743 | -0.692502 |
| H | 0.121088 | 0.722188 | -3.586056 |
| H | 0.048993 | -1.613400 | -1.661307 |

68

Complex K

| | | | |
|---|---|---|---|
| Mo | 0.018074 | -0.479393 | -0.152869 |
| P | 2.366713 | 0.270078 | 0.145864 |
| P | -2.399785 | -0.231123 | 0.376441 |
| N | -0.216502 | 1.663388 | -0.287439 |
| N | 2.148694 | 1.967282 | -0.226691 |
| N | -2.590597 | 1.435838 | -0.124692 |
| C | 0.889938 | 2.468619 | -0.503824 |
| C | 0.745943 | 3.782866 | -0.968263 |
| C | -0.526541 | 4.282779 | -1.181689 |
| C | -1.649722 | 3.516010 | -0.910011 |
| C | -1.480700 | 2.206204 | -0.446777 |
| C | 3.310021 | 0.326556 | 1.763470 |
| C | 2.443992 | 0.977755 | 2.856314 |
| C | 3.444529 | -0.451208 | -1.221557 |
| C | 4.910720 | 0.016289 | -1.228869 |
| C | -4.106147 | 0.599406 | 2.598526 |
| C | -3.241785 | -1.791199 | 2.586403 |
| C | -3.469343 | -1.260502 | -0.796379 |
| C | -4.996909 | -1.215819 | -0.601440 |
| H | 1.608963 | 4.391745 | -1.186066 |
| H | -0.648511 | 5.292364 | -1.561604 |
| H | -2.632389 | 3.928973 | -1.071568 |
| H | 3.051106 | 1.131790 | 3.753949 |
| H | 1.606341 | 0.329387 | 3.129107 |
| H | 2.051366 | 1.952844 | 2.551444 |
| H | 5.427868 | -0.480400 | -2.056345 |
| H | 5.440566 | -0.255888 | -0.311374 |
| H | 5.015969 | 1.090688 | -1.387227 |
| H | -4.253273 | 0.490385 | 3.678003 |
| H | -5.054150 | 0.342016 | 2.117276 |
| H | -3.884583 | 1.651398 | 2.406341 |

| | | | |
|---|---|---|---|
| H | -3.363949 | -1.824886 | 3.673654 |
| H | -2.418930 | -2.459405 | 2.328402 |
| H | -4.166539 | -2.168313 | 2.142935 |
| H | -5.455432 | -1.850768 | -1.366602 |
| H | -5.426499 | -0.220577 | -0.719614 |
| H | -5.301206 | -1.614270 | 0.369266 |
| C | -2.979438 | -2.723982 | -0.831215 |
| H | -1.916672 | -2.802616 | -1.062589 |
| H | -3.166980 | -3.241994 | 0.112231 |
| H | -3.532774 | -3.253337 | -1.613458 |
| C | 3.355760 | -1.991440 | -1.242049 |
| H | 2.327482 | -2.348793 | -1.315586 |
| H | 3.899403 | -2.356189 | -2.119585 |
| H | 3.814270 | -2.446150 | -0.360704 |
| C | 3.837929 | -1.051140 | 2.201890 |
| H | 4.568494 | -1.462091 | 1.501506 |
| H | 4.341486 | -0.939007 | 3.167656 |
| H | 3.026101 | -1.770453 | 2.325598 |
| H | -2.053891 | -0.012636 | 2.700511 |
| C | -2.961756 | -0.333639 | 2.173245 |
| H | -3.224978 | -0.800777 | -1.763171 |
| H | 2.944587 | -0.065938 | -2.119038 |
| H | 4.174553 | 0.974735 | 1.573753 |
| Br | 0.290588 | -2.166290 | 1.754269 |
| C | 3.283903 | 2.900211 | -0.305241 |
| H | 3.089923 | 3.792236 | 0.297441 |
| H | 4.175468 | 2.421098 | 0.090606 |
| H | 3.484421 | 3.198135 | -1.339681 |
| C | -3.913364 | 2.063560 | -0.282606 |
| H | -4.129384 | 2.267232 | -1.336906 |
| H | -4.675846 | 1.396997 | 0.104180 |
| H | -3.973693 | 2.997157 | 0.284110 |
| O | 0.108967 | 0.955234 | -3.605619 |
| O | 1.540855 | 1.201997 | -3.665721 |
| O | 0.014720 | -1.189897 | -1.685918 |
| H | 1.690687 | 1.139012 | -4.622821 |
| H | 0.094346 | 0.079608 | -3.174695 |

65
Complex TSFL

| | | | |
|---|---|---|---|
| Mo | 0.098024 | -0.482178 | -0.343372 |
| P | 2.438052 | 0.354319 | -0.001733 |
| P | -2.386784 | -0.176715 | -0.012006 |
| N | -0.180549 | 1.709583 | -0.168381 |
| N | 2.102822 | 2.012598 | 0.428890 |
| N | -2.431373 | 1.381536 | -0.806554 |
| C | 0.866935 | 2.562747 | 0.111816 |
| C | 0.700046 | 3.951554 | 0.067713 |
| C | -0.528883 | 4.474693 | -0.315762 |
| C | -1.582367 | 3.636637 | -0.636401 |
| C | -1.394804 | 2.250388 | -0.539288 |
| C | 3.643730 | -0.244751 | 1.305559 |
| C | 3.200602 | 0.120205 | 2.733394 |
| C | 3.299506 | 0.418080 | -1.681680 |
| C | 4.439901 | 1.446030 | -1.769564 |
| C | -4.239080 | 1.152918 | 1.762435 |
| C | -3.420634 | -1.159248 | 2.457495 |
| C | -3.462081 | -1.334033 | -1.014291 |
| C | -4.984102 | -1.253309 | -0.799105 |
| H | 1.519347 | 4.615531 | 0.294816 |

| | | | |
|---|---|---|---|
| H | -0.662489 | 5.551053 | -0.359465 |
| H | -2.546377 | 4.041313 | -0.907926 |
| H | 3.980001 | -0.200508 | 3.431893 |
| H | 2.279313 | -0.401663 | 3.001015 |
| H | 3.044584 | 1.190412 | 2.881457 |
| H | 4.872065 | 1.399249 | -2.774198 |
| H | 5.248943 | 1.231013 | -1.063744 |
| H | 4.098654 | 2.470594 | -1.613095 |
| H | -4.510223 | 1.325485 | 2.809330 |
| H | -5.131990 | 0.782530 | 1.252367 |
| H | -3.966359 | 2.120410 | 1.333627 |
| H | -3.621650 | -0.920541 | 3.506779 |
| H | -2.600067 | -1.878421 | 2.435623 |
| H | -4.318234 | -1.635551 | 2.056006 |
| H | -5.471860 | -1.865504 | -1.564724 |
| H | -5.389266 | -0.242734 | -0.882554 |
| H | -5.280562 | -1.656684 | 0.171592 |
| C | -2.954678 | -2.780892 | -0.839709 |
| H | -1.906182 | -2.879715 | -1.128339 |
| H | -3.073476 | -3.140580 | 0.186317 |
| H | -3.541995 | -3.436663 | -1.489944 |
| C | 3.769874 | -0.961288 | -2.179831 |
| H | 3.018433 | -1.741648 | -2.048192 |
| H | 3.977292 | -0.885746 | -3.251738 |
| H | 4.698661 | -1.266587 | -1.690408 |
| C | 3.879531 | -1.764711 | 1.187945 |
| H | 4.259652 | -2.066584 | 0.212191 |
| H | 4.624258 | -2.055498 | 1.935403 |
| H | 2.963312 | -2.322248 | 1.392798 |
| H | -2.216380 | 0.597234 | 2.219532 |
| C | -3.078829 | 0.144640 | 1.711372 |
| H | -3.207589 | -1.027797 | -2.035110 |
| H | 2.479566 | 0.747194 | -2.331972 |
| H | 4.589790 | 0.264683 | 1.077578 |
| Br | 0.143243 | -1.875105 | 1.742044 |
| C | 3.159911 | 2.933933 | 0.881676 |
| H | 2.835220 | 3.461748 | 1.783056 |
| H | 4.057352 | 2.369857 | 1.122495 |
| H | 3.421491 | 3.667257 | 0.113113 |
| C | -3.571879 | 1.868308 | -1.597023 |
| H | -3.203125 | 2.490445 | -2.414934 |
| H | -4.089015 | 1.021357 | -2.040955 |
| H | -4.286182 | 2.441330 | -0.995063 |
| O | -0.822703 | -0.374136 | -2.125279 |
| O | 0.670045 | -1.438217 | -1.686895 |

65

Complex L

| | | | |
|---|---|---|---|
| Mo | 0.072436 | -0.488397 | 0.026038 |
| P | 2.446138 | 0.447247 | 0.012453 |
| P | -2.454630 | -0.032303 | 0.036898 |
| N | -0.147475 | 1.927785 | -0.096703 |
| N | 2.209497 | 2.148374 | 0.095394 |
| N | -2.494225 | 1.644421 | -0.365053 |
| C | 0.939598 | 2.734878 | 0.010434 |
| C | 0.819900 | 4.132591 | 0.024881 |
| C | -0.447442 | 4.687260 | -0.062304 |
| C | -1.565064 | 3.874744 | -0.178468 |
| C | -1.380413 | 2.485211 | -0.202242 |
| C | 3.678323 | -0.092983 | 1.325322 |

| | | | |
|---|---|---|---|
| C | 3.705627 | 0.779236 | 2.591784 |
| C | 3.178265 | 0.050814 | -1.671844 |
| C | 4.458263 | 0.819332 | -2.039653 |
| C | -4.689474 | 0.327676 | 1.848301 |
| C | -3.305352 | -1.823230 | 2.011370 |
| C | -3.282261 | -0.961415 | -1.385038 |
| C | -4.815226 | -1.113981 | -1.314792 |
| H | 1.683857 | 4.773439 | 0.106205 |
| H | -0.565666 | 5.765837 | -0.036026 |
| H | -2.550010 | 4.312760 | -0.229167 |
| H | 4.455378 | 0.362676 | 3.272050 |
| H | 2.741478 | 0.765595 | 3.101208 |
| H | 3.982625 | 1.815943 | 2.397612 |
| H | 4.789503 | 0.487671 | -3.029076 |
| H | 5.281613 | 0.619185 | -1.345658 |
| H | 4.300699 | 1.897701 | -2.097519 |
| H | -5.013948 | 0.161807 | 2.880698 |
| H | -5.448712 | -0.106101 | 1.194549 |
| H | -4.663097 | 1.408253 | 1.690290 |
| H | -3.608256 | -1.945666 | 3.055667 |
| H | -2.314827 | -2.275029 | 1.905255 |
| H | -4.015113 | -2.384908 | 1.397721 |
| H | -5.155881 | -1.545837 | -2.261181 |
| H | -5.361760 | -0.180336 | -1.171466 |
| H | -5.112100 | -1.803310 | -0.520578 |
| C | -2.645616 | -2.353320 | -1.594221 |
| H | -1.579165 | -2.298807 | -1.810865 |
| H | -2.795139 | -3.004955 | -0.728986 |
| H | -3.135383 | -2.827685 | -2.450344 |
| C | 3.378720 | -1.467241 | -1.847298 |
| H | 2.490137 | -2.049493 | -1.590038 |
| H | 3.607357 | -1.667606 | -2.898432 |
| H | 4.222434 | -1.835015 | -1.255978 |
| C | 3.428718 | -1.571070 | 1.697431 |
| H | 3.391034 | -2.237392 | 0.833811 |
| H | 4.248801 | -1.907837 | 2.338899 |
| H | 2.495027 | -1.677607 | 2.254304 |
| H | -2.609391 | 0.172642 | 2.361443 |
| C | -3.306083 | -0.319572 | 1.674100 |
| H | -3.014622 | -0.332631 | -2.243762 |
| H | 2.368737 | 0.365749 | -2.341411 |
| H | 4.650106 | -0.022010 | 0.818634 |
| Br | 0.047059 | 0.242843 | 2.578935 |
| C | 3.374885 | 3.047519 | 0.173215 |
| H | 3.319336 | 3.674527 | 1.067693 |
| H | 4.287646 | 2.458457 | 0.229819 |
| H | 3.438956 | 3.686645 | -0.713075 |
| C | -3.745366 | 2.278056 | -0.820395 |
| H | -3.547811 | 2.892172 | -1.703074 |
| H | -4.461122 | 1.511191 | -1.100866 |
| H | -4.200760 | 2.899772 | -0.042986 |
| O | 0.044341 | -0.410794 | -1.699770 |
| O | 0.229784 | -2.148367 | 0.404451 |
